# Comprehensive Evaluation of Cleavable Bioorthogonal Probes for Site-Specific O-GlcNAc Proteomics

**DOI:** 10.1016/j.mcpro.2025.101064

**Published:** 2025-08-28

**Authors:** Chunyan Hou, Hemeng Zhang, Jingtao Deng, Xiaoxin Wang, Stephen Byers, Moshe Levi, Daniel T.S. Pak, Kelley W. Moremen, Huadong Pei, Gerald W. Hart, Junfeng Ma

**Affiliations:** 1 Department of Oncology, Lombardi Comprehensive Cancer Center, Georgetown University Medical Center, Washington, District of Columbia, USA; 2 Department of Biochemistry and Molecular & Cellular Biology, Georgetown University Medical Center, Washington, District of Columbia, USA; 3 Department of Pharmacology and Physiology, Georgetown University Medical Center, Washington, District of Columbia, USA; 4 Complex Carbohydrate Research Center, University of Georgia, Athens, Georgia, USA

**Keywords:** O-GlcNAc, proteomics, PC-biotin-alkyne, DADPS-biotin-alkyne, Dde-biotin-alkyne, Diazo-biotin-alkyne, chemoenzymatic labeling

## Abstract

O-linked β-N-acetylglucosamine (O-GlcNAc) modification (*i.e.*, O-GlcNAcylation) on proteins is an essential modification in physiology and pathology. Although O-GlcNAcylation is functionally critical, its analysis has been challenging. Despite the existence of a number of methods developed in the past years, which one(s) might have the best performance is largely unclear. To that end, we conducted a rigorous comparison of several cleavable bioorthogonal biotin-alkyne probes which showed promise for sensitive O-GlcNAc proteomics. In brief, we developed chemoenzymatic labeling/click chemistry-based analytical workflows for O-GlcNAc proteomics by utilizing four cleavable bioorthogonal probes, including photocleavabe-biotin-alkyne (PC-biotin-alkyne), dialkoxydiphenylsilane-biotin-alkyne (DADPS-biotin-alkyne); 1-(4,4-dimethyl-2,6-dioxocyclohex-1-ylidene)ethyl-biotin-alkyne (Dde-biotin-alkyne), and diazobenzene-biotin-alkyne (Diazo-biotin-alkyne). The analytical performance of these probes was evaluated with synthetic O-GlcNAc peptides and then benchmarked by using mouse brain lysates for O-GlcNAc proteomics. Besides providing valuable technical insights into O-GlcNAc proteomics methods, our work yielded an unprecedented O-GlcNAc proteome depth in the mouse brain. In total, 2906 O-GlcNAc sites were unambiguously assigned on 878 proteins. Among them, 1611 sites were newly identified, including 138 O-GlcNAcylated tyrosine residues. Our work will help guide the selection/development of O-GlcNAc proteomics methods for future studies, provide an invaluable resource for functional elucidation of protein O-GlcNAcylation in brain biology, and yield critical insights into tyrosine O-GlcNAcylation.

## Highlights

• Head-to-head comparison of cleavable bioorthogonal probes for O-GlcNAc proteomics.
• >2900 unambiguous O-GlcNAc sites on 878 proteins identified from mouse brain.
• Tyrosine O-GlcNAcylation on 138 residues of 90 proteins found in mouse brain.

## Graphical Abstract

**Figure:**
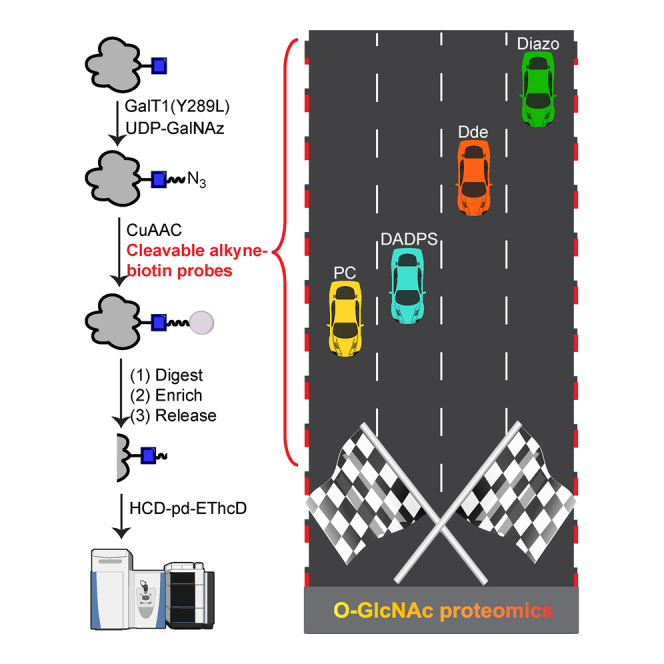

## In Brief

O-linked β-N-acetylglucosamine (O-GlcNAc) modification (*i.e.*, O-GlcNAcylation), despite its functional importance, suffers from severe technical challenges to analyze. We performed systematic evaluation of several cleavable bioorthogonal probes for site-specific O-GlcNAc proteomics. Our work provides not only valuable technical insights to O-GlcNAc proteomics methods, but also a comprehensive O-GlcNAc proteome dataset of mouse brain (including tyrosine O-GlcNAcylation).

O-linked β-N-acetylglucosamine (O-GlcNAc) addition (O-GlcNAcylation) is a fundamentally important post-translational modification on proteins (1, 2). Distinct from the traditional N-glycosylation and mucin-type O-glycosylation, O-GlcNAcylation almost exclusively modifies intracellular proteins (3). O-GlcNAcylation is predominantly present in the nucleus and cytoplasm and is also found in mitochondria (4, 5, 6). Besides the classic serine (Ser) and threonine (Thr) residues, we recently identified tyrosine (Tyr) as a new target for protein O-GlcNAcylation (7). O-GlcNAc addition is catalyzed by O-GlcNAc-transferase (OGT) and removed by O-GlcNAcase (OGA). To date, >20% of the human proteome (>4000 proteins) are known to be O-GlcNAc modified with at least one site (8, 9). O-GlcNAcylation has been found in many species, ranging from viruses and bacteria to plants and mammals (10). It is now clear that O-GlcNAcylation plays critical roles in virtually all biochemical processes examined (including transcription, translation, the cell cycle, metabolism and signaling) (11, 12, 13, 14, 15, 16, 17).

The brain is one of the tissues that shows the highest expression of OGT/OGA and O-GlcNAcylation levels (10, 18, 19). Neuron-specific deletion of OGT leads to neonatal lethality, likely due to abnormal neuronal development (20). O-GlcNAcylation is directly linked to a broad range of brain functions, including learning, memory, satiety, movement, and others (21, 22, 23). Protein O-GlcNAcylation is not only important for brain physiology but is also associated with several neurodegenerative disorders (*e.g.*, Alzheimer’s disease and Parkinson’s disease) (24, 25). For example, the presence of O-GlcNAcylation on proteins, such as microtubule-associated protein Tau and β-amyloid precursor protein APP, in brain samples were reported shortly after the discovery of protein O-GlcNAcylation (26, 27). In addition, overall compromised O-GlcNAcylation levels were observed in brain and pluripotent stem cell-derived neurons from patients with Alzheimer’s disease (28, 29). Increased O-GlcNAcylation on Tau and α-synuclein (α-Syn) can also influence the formation of toxic aggregates which are associated with neurodegeneration (30, 31, 32, 33). Thus, modulating O-GlcNAc signaling may represent a promising neuroprotective strategy to treat neurodegenerative diseases. To date, a number of inhibitors targeting OGA have been tested in clinical trials (34, 35, 36, 37).

Site-specific O-GlcNAc characterization is instrumental in the dissection of O-GlcNAc signaling and its functional consequences in different biomedical scenarios. Significant progress has been made with technological advances in high-throughput methods, especially tandem mass spectrometry (MS/MS)-based proteomics in recent years (38, 39, 40, 41). However, large-scale O-GlcNAc site mapping remains a daunting challenge—it is still far from routine to identify a few thousand or even several hundred O-GlcNAc proteins/sites in individual studies. Given that the substoichiometric nature of O-GlcNAcylation appears to be a key hurdle, selective and sensitive enrichment for O-GlcNAc proteins/peptides is indispensable. By leveraging the unique biochemical properties of O-GlcNAc, two categories of enrichment materials/methods have been developed: one is affinity enrichment, exemplified by using antibodies (42, 43), lectins (44, 45, 46, 47, 48, 49, 50, 51), and hydrophilic interaction chromatography (52, 53); another is chemical/biochemical derivatization, e.g., alkaline β-elimination followed by Michael addition (54, 55, 56), and chemoenzymatic-/metabolic-labeling followed by click chemistry (57, 58, 59, 60, 61, 62, 63, 64, 65, 66, 67, 68, 69, 70, 71, 72, 73). Although affinity-based enrichment materials/methods provide a convenient tool, their affinity and specificity towards the monosaccharide O-GlcNAc moiety have been notoriously weak, rendering poor enrichment efficiency (generally <5%) (74). In contrast, (bio)chemical derivatization serves as a promising alternative. The metabolic labeling approach (*e.g.*, by using azido-derived analogs such as N-azidoacetylgalactosamine-tetraacylated (Ac4GalNAz) (75, 76, 77), utilizing the UDP-GlcNAc salvage pathways in cells) simplifies the enrichment workflow somewhat, but it is not applicable to tissue samples. Chemoenzymatic labeling, in conjunction with click chemistry, particularly by using cleavable biotin-alkyne probes, represents a promising enrichment method for sensitive O-GlcNAc proteomics, regardless of sample sources. A series of cleavable biotin-alkyne probes have been developed and applied for O-GlcNAc proteomics, including photocleavabe-biotin-alkyne (PC-biotin-alkyne) (7, 58, 60, 61, 65, 68, 70, 71), 1-(4,4-dimethyl-2,6-dioxocyclohex-1-ylidene)ethyl-biotin-alkyne (Dde-biotin-alkyne) (62), dialkoxydiphenylsilane-biotin-alkyne (DADPS-biotin-alkyne) (64, 71), disulfide-biotin-dibenzocyclooctyne (Disulfide Biotin DBCO) (59), and diazobenzene-biotin-alkyne (Diazo-biotin-alkyne) (72, 73). Although there are multiple cleavable biotin-alkyne probes, their performance for O-GlcNAc proteomics has not been investigated systematically.

The goal of the present study was to perform a rigorous comparison of several cleavable biotin-alkyne probes (including PC-biotin-alkyne, DADPS-biotin-alkyne, Dde-biotin-alkyne, and Diazo-biotin-alkyne) for chemoenzymatic labeling-based O-GlcNAc proteomics in a site-specific manner. After being evaluated with synthetic O-GlcNAc peptides, the probes were benchmarked for O-GlcNAc proteomics of mouse brain lysates. In total, 2906 O-GlcNAc sites were unambiguously identified, representing the biggest dataset of any biological samples (including brain) characterized in one study to date. Among the 1611 sites newly reported, 138 tyrosine sites were O-GlcNAcylated, suggesting that endogenous Tyr O-GlcNAcylation is widespread in murine brain (even without external stimulation). Collectively, our work not only provides technical insights into the O-GlcNAc proteomics methods but also serves as a comprehensive resource for site-specific functional studies of protein O-GlcNAcylation in brain physiology.

## Experimental Procedures

### Experimental Design and Rationale

The lack of sensitive and robust methods for O-GlcNAc proteomics has been a major hurdle for many years, largely due to the limited efficiency of enrichment methods. Despite the recent emergence of a panel of methods, their performance was almost exclusively illustrated by the analysis of selected specific samples. We reasoned that a systematic evaluation of current methods would facilitate further advances of O-GlcNAc proteomics and its biomedical applications.

Recently, we performed a head-to-head comparison of several affinity-based enrichment materials/methods (including the OGA mutant CpOGA^D298N^, lectin AANL6, and the PTMScan O-GlcNAc [GlcNAc-S/T] motif kit) for O-GlcNAc proteomics of human pancreatic cancer-derived PANC-1 cells (74). Moreover, we prepared nitro-oxide-grafted nanospheres with enhanced hydrogen bonding interaction for O-GlcNAc proteomics (53), yielding substantially more O-GlcNAc sites from the similar cell lysate samples after analysis with the similar set of nanoUPLC-MS/MS instruments and experimental settings (although this method was not directly included in the head-to-head comparison study). In addition, built upon enrichment by incorporating chemoenzymatic labeling with PC-biotin-alkyne-based click chemistry, we developed an integrated workflow and benchmarked its performance for ultradeep O-GlcNAc proteomics of PANC-1 cells (7). Our data showed that, in comparison to these affinity-based enrichment materials/methods, chemoenzymatic labeling followed by PC-biotin-alkyne-based click chemistry has some striking advantages, such as the use of much lower amounts of starting material, much higher enrichment selectivity, and more O-GlcNAc sites/proteins identified (Supplemental Table S1). Prompted by these findings, we proposed to further evaluate the performance of the chemoenzymatic labeling/click chemistry-based method. Specifically, we set out to compare four cleavable biotin-alkyne probes (including PC-biotin-alkyne, DADPS-biotin-alkyne, Dde-biotin-alkyne, and Diazo-biotin-alkyne), which are commercially available and have been exploited for O-GlcNAc proteomics. Given that different cleavable biotin-alkyne probes might have different performance, we aimed to conduct a head-to-head comparison of the four cleavable biotin-alkyne reagents for site-specific O-GlcNAc proteomics. We first set up the methods using synthetic O-GlcNAc peptides, then we compared the analytical performance of the four cleavable biotin-alkyne reagents for O-GlcNAc proteomics by analyzing mouse brain samples.

### Labeling of Standard O-GlcNAc Peptides

Standard peptides TAPTS(O-GlcNAc)TIAPG (in which S5 is O-GlcNAcylated) and YSPT(O-GlcNAc)SPSK (in which T4 is O-GlcNAcylated) were custom-synthesized by AnaSpec, Inc. Another peptide AGY(O-GlcNAc)SQGATQYTQAQQTR (in which Y3 is O-GlcNAcylated) was synthesized in our lab, as described previously (7). A mixture of the three O-GlcNAcylated peptides was prepared in 50 μl of 20 mM HEPES buffer (pH 7.9). Subsequently, 11 μl of 100 mM MnCl_2_, 12.5 μl of 0.5 mM uridine 5′-diphospho-*N*-acetylazidogalactosamine disodium salt (UDP-GalNAz; BioChemSyn), and 4 μg of recombinant GFP-GalT1 (Y289L, prepared by following the procedure described previously (78)) were added. The mixture was incubated at 4 °C overnight, followed by desalting using a C18 spin column (BioPureSPN Mini, The Nest Group). The peptides were dried, dissolved in 275 μl of 50 mM HEPES buffer (pH 7.9), and split into equal parts for copper-catalysed azide–alkyne cycloaddition (CuAAC) with the four cleavable biotin alkynes (Cat# CCT-1454, Vector Laboratories) in parallel. Briefly, 1 μl of 10 mM PC-, DADPS-, Dde-, or Diazo-biotin-alkyne, 3 μl of 10 mM CuSO_4_-BTTAA (1:2, measured as CuSO_4_; Vector Laboratories), and 5 μl of 50 mM sodium ascorbate were added in order. The mixture was incubated at room temperature for 2 h. Next, 0.5 ml of PBS buffer was added along with 120 μl of high-capacity NeutrAvidin agarose slurry (Thermo Fisher Scientific) and incubated for 3 h, followed by sequential washing with cold PBS, water, 20% methanol, and 70% methanol. The enriched peptides conjugated with PC-biotin alkyne were released in 400 μl of 0.1% formic acid (FA) by irradiation at 365 nm (UVP XX-15L UV Bench Lamp, Analytik Jena) for 1 h. The peptides conjugated with DADPS-, Dde-, and Diazo-biotin-alkynes were released by incubation in 400 μl of 10% FA, 2% hydrazine, and freshly prepared 25 mM Na_2_S_2_O_4_ aqueous solution for 1 h, respectively (by referring to previous publications (62, 64, 73)). The released peptides were then cleaned up using a C18 spin column, dried, and kept at −20 °C before nanoUPLC-MS/MS analysis.

### Protein Extraction From Mouse Brain

Mice were obtained from Jackson Laboratories and maintained according to the National Institute of Health Guide for Care and Use of Laboratory Animals, with the approval by the Institutional Animal Care and Use Committee of Georgetown University (with the protocol # 2017-0059), as described previously (79). In brief, C57BL/6 wild-type mice were raised in an animal care facility with 12/12-h light-dark cycle, with access to a regular chow diet and water *ad libitum*. Three-month old mice were euthanized by carbon dioxide inhalation, and the whole brain excised. Mouse brain was washed with cold PBS buffer before being cut into small pieces on ice. Subsequently, 0.5 ml of a lysis buffer (containing 5% sodium dodecyl sulfate (SDS), 50 mM triethylammonium bicarbonate (TEAB), 1 × cOmplete EDTA-free protease inhibitor cocktail, 75 mM NaCl, 1 mM EDTA and 50 μM PUGNAc (MilliporeSigma)) was added. Tissue samples were then homogenized and sonicated on ice. Next, 5 μl of 0.1 M MgCl_2_ and 5 μl of Benzonase nuclease (MilliporeSigma) were added followed by an incubation at room temperature for 20 min. The lysate was cleared by centrifugation at 14,000*g* at 4 °C for 20 min. The concentration of proteins in the supernatant was determined by BCA assay.

### Enrichment of O-GlcNAc Peptides From Mouse Brain Extracts

Four milligrams of proteins extracted from the mouse brain were used for each enrichment with the biotin alkynes, using a procedure similar to what we described previously (7). Briefly, proteins were first reduced with 20 mM 1,4-dithiothreitol (DTT), then alkylated with 40 mM iodoacetamide (IAA). After methanol/chloroform precipitation, proteins were resuspended in 400 μl of 20 mM HEPES buffer (pH 7.9) containing 1% SDS. Next, 490 μl of water, 800 μl of labeling buffer (125 mM NaCl, 5% NP-40, 50 mM HEPES, pH 7.9), 105 μl of 100 mM MnCl_2_, 100 μl of 0.5 mM UDP-GalNAz, 10 μg GFP-GalT1 (Y289L), 0.5 μl of recombinant PNGase F (VWR), and 1 μl of thermosensitive alkaline phosphatase (TSAP, Cat# M9910, Promega) were added and incubated at 4 °C overnight. Then an additional 0.5 μl of TSAP was added, followed by incubation at 37 °C for two more hours. The proteins were precipitated again and resuspended in 1 ml of HEPES buffer (pH 7.9) containing 0.5% SDS. To the solution, 11 μl of 10 mM PC-, DADPS-, Dde-, or Diazo-biotin-alkyne, 33 μl of 10 mM CuSO_4_-BTTAA (1:2), and 55 μl of 50 mM sodium ascorbate were added and incubated at room temperature for 2 h. After precipitation, the proteins were dissolved in 0.8 ml of 50 mM TEAB buffer (pH 8) containing 8 M urea, diluted by 50 mM TEAB buffer, and digested with trypsin (w/w = ∼30:1) at 37 °C overnight. O-GlcNAc peptides in the digests were enriched and released following the same protocol described in the previous section for the labeling of standard O-GlcNAc peptides.

### nanoUPLC-MS/MS

The nanoUPLC-MS/MS analysis was performed on a nanoAcquity UPLC system (Waters) coupled to an Orbitrap Fusion Lumos mass spectrometer (Thermo Fisher) with similar settings described previously (7) with slight modifications. Dried samples were dissolved in 0.1% FA and loaded onto a C18 Trap column (Waters Acquity UPLC M-Class Trap, Symmetry C18, 100 Å, 5 μm, 180 μm × 20 mm) at 10 μl/min for 4 min. Separation was carried out on an analytical column (Waters Acquity UPLC M-Class, Peptide BEH C18, 300 Å, 1.7 μm, 75 μm × 150 mm) at a column temperature of 45 °C and a flow rate of 400 nl/min, using 60- and 300-min gradients for standard peptides and mouse brain samples, respectively. The 60-min gradient of buffer A (2% ACN, 0.1% formic acid) and buffer B (0.1% formic acid in ACN) was as follows: 1% buffer B at 0 min, 5% buffer B at 1 min, 30% buffer B at 40 min, 50% buffer B at 50 min, 90% buffer B at 52 min, 90% buffer B at 60 min. The 300-min gradient was 1% buffer B at 0 min, 5% buffer B at 1 min, 30% buffer B at 210 min, 45% buffer B at 260 min, 90% buffer B at 270 min, 90% buffer B at 290 min, 1% buffer B at 290.1 min, and 1% buffer B at 300 min. The MS data were acquired in data-dependent acquisition (DDA) mode by an Orbitrap Fusion Lumos mass spectrometer with an ion spray voltage of 2.4 kV and an ion transfer temperature of 275 °C. The mass spectra were recorded with Xcalibur 4.7. The MS parameters were set as below: Detector Type: Orbitrap; Orbitrap Resolution: 120,000; Scan Range: 350-1800 m/z; RF Lens: 30%; AGC Target: Standard; Maximum Injection Time Mode: Auto; Microscans: 1. Charge state: 2 to 8; Cycle Time: 3 s.

EThcD without dynamic exclusion was used for standard peptides, whereas HCD product-dependent EThcD (HCD-pd-EThcD) with a dynamic exclusion duration of 40 s was applied for MS/MS acquisition of mouse brain samples. In HCD-pd-EThcD mode, EThcD was triggered by the oxonium ions of HexNAc (m/z 126.055, 138.055, 144.066, 168.065, 186.076, and 204.086), as well as the major fragments resulting from the tags—m/z 300.130 and 503.210 for both PC- and Dde-biotin-alkyne, m/z 329.146 and 532.225 for DADPS-biotin-alkyne, and m/z 507.220 and 710.299 for Diazo-biotin-alkyne—observed in HCD scans. MS/MS parameters were set as below: Isolation Mode: Quadrupole; Isolation Window: 1.6 m/z; Stepped collision energy of 22.5, 30, 37.5% for HCD was used. Detector Type: Orbitrap; Resolution: 30,000; Normalized AGC Target: 200%. Supplemental activation (SA) collision energy of EThcD was set as 30%.

### Data Analysis

Mass spectrometric data files for synthetic peptides were processed with Proteome Discoverer (PD, version 2.4; Thermo Fisher Scientific) with Sequest HT, as described previously (53, 74, 80). Proteome Discoverer (PD, version 2.4; Thermo Fisher Scientific) with Sequest HT and MaxQuant (MQ, version 2.3.0.0, Max Planck Institute of Biochemistry) were used to process the raw data files of mouse brain samples using similar parameters as those described previously (7). UniProt *Mus musculus* database (TaxID: 10090, downloaded on February 2, 2023; 17137 sequences) was used for database searching. Raw files of each biotin alkyne were searched as a single batch. The false-discovery rate (FDR) was determined by using a target-decoy search strategy. The decoy-sequence database contains each sequence in reverse orientation, enabling FDR estimation. In PD, strict and relaxed target FDRs for highly and medium confident peptide/protein hits were lower than 1% and 5%, respectively. In MQ, FDR was set at 1% for both PSM and protein. Full digestion mode was selected for trypsin at Lys and Arg, allowing a maximum of two missed cleavages. Mass additions of 709.292 Da and 531.217 Da on Ser/Thr/Tyr/Asn were searched for Diazo- and DADPS-treated samples, respectively, with 502.202 Da applied to both Dde- and PC-treated samples. As many as five modifications were allowed per peptide. Other settings for PD and MQ were kept as the default unless otherwise specified. The precursor mass tolerance was set at 10 ppm, whereas the fragment-mass tolerance was set at 0.02 Da. Carbamidomethylation of cysteines (+57.0215 Da) was set as a fixed modification, and variable modifications of deamidation (N, +0.984 Da), methionine oxidation (M, +15.9949 Da), acetyl (N-terminus, +42.011 Da), and loss (N-terminus M, −131.040 Da), or loss + acylation (N-terminus M, −89.030 Da) were allowed. The modification sites identified by Sequest HT were further filtered by IMP-ptmRS with a site probability ≥0.75. When using MQ, carbamidomethylation of cysteines was set as a fixed modification, and the variable modifications included methionine oxidation, asparagine deamidation, and acetyl (N-terminus). A localization probability threshold of 0.75 was used to distinguish unambiguous and ambiguous sites. The raw files of standard peptide samples were searched against a customized FASTA file containing the corresponding peptide sequences without cleavage using PD with a fixed value PSM validator (delta Cn, which is the normalized score difference between the currently selected PSM and the highest-scoring PSM for that spectrum, better than or equal to 0.05). The MS level chromatograms of labeled standard peptides were extracted at the theoretical m/z with a mass tolerance of 5 ppm using Thermo Scientific FreeStyle 1.8 SP2.

### Statistics

Labeling of standard O-GlcNAc peptides was performed in triplicate for each biotin alkyne reagent, with labeled intensity quantified at the MS1 level based on peak area. Mouse brain lysates pooled from six mice were used for four enrichments per biotin alkyne reagent, and each sample was analyzed in duplicate by nanoUPLC-MS/MS. All eight runs per biotin alkyne reagent were searched as a single batch. Statistical mean comparisons between box blots were assessed using a *t* test.

## Results

### Development of Workflows for O-GlcNAc Peptides

**Fig. 1:**
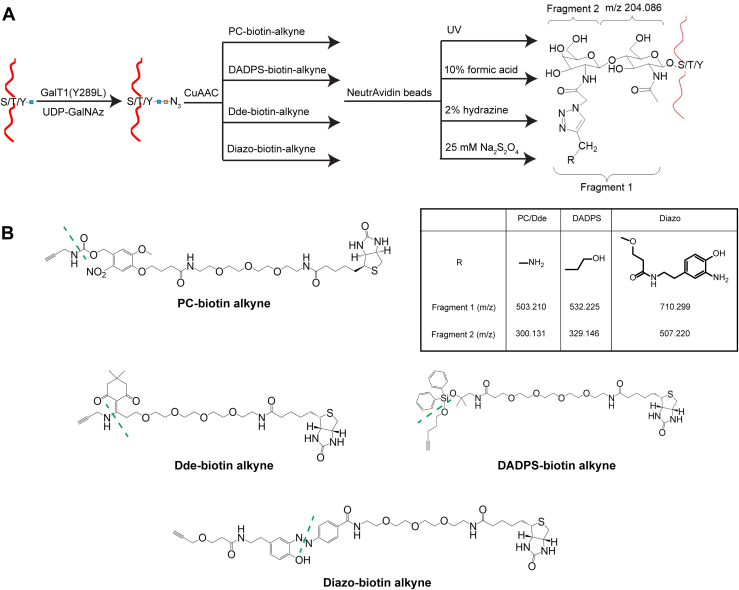
**Analysis of O-GlcNAc peptides by using four cleavable biotin-alkyne probes.***A*, schematic diagram for O-GlcNAc analysis of peptides, which involves GalT1(Y289L)-mediated chemoenzymatic labeling, click chemistry with cleavable biotin-alkyne probes, capture with neutravidin beads, and release of tagged O-GlcNAc peptides. The released peptides are then subjected to tandem mass spectrometry in EThcD mode, with the data files analyzed by Proteome Discoverer. *B*, structures of four cleavable biotin-alkyne probes, *i.e.*, PC-biotin-alkyne, DADPS-biotin-alkyne, Dde-biotin-alkyne, and Diazo-biotin-alkyne. Cleavage sites are indicated in *dashed green lines*.

Three standard O-GlcNAc peptides, including YSPT(O-GlcNAc)SPSK, TAPTS(O-GlcNAc)TIAPG, and AGY(O-GlcNAc)SQGATQYTQAQQTR, were used to develop the workflows and assess their performance for O-GlcNAc analysis (Fig. 1*A*). In brief, peptides were subjected to GalT1(Y289L)-mediated chemoenzymatic labeling and then click chemistry with four cleavable biotin-alkyne probes, including PC-biotin-alkyne, DADPS-biotin-alkyne, Dde-biotin-alkyne, and Diazo-biotin-alkyne (Fig. 1*B*). Biotin-tagged O-GlcNAc peptides were captured on neutravidin beads and then released in specific cleavage conditions. The released peptides were then analyzed by nanoUPLC-MS/MS in electron-transfer/higher-energy collision dissociation (EThcD) mode.

**Fig. 2:**
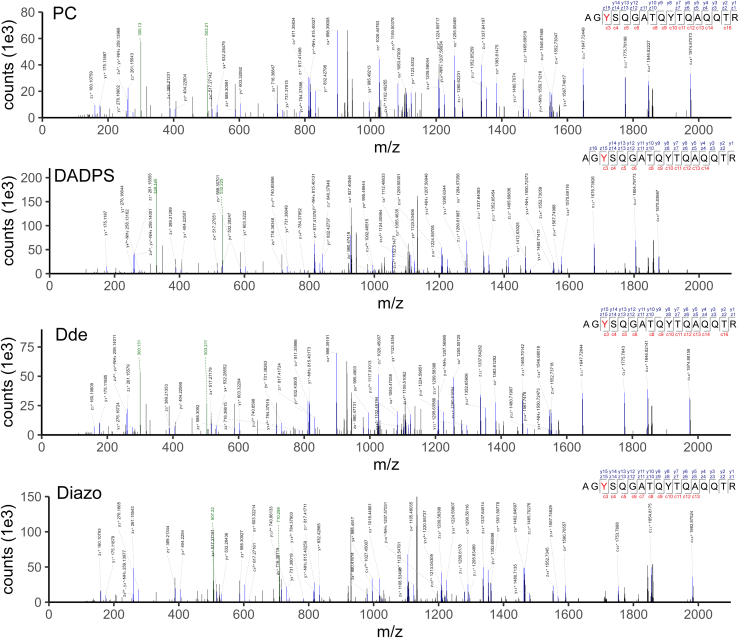
**Representative mass spectra of synthetic O-GlcNAc peptides after enrichment, with tyrosine O-GlcNAcylated peptide ‘AGY(O-GlcNAc)SQGATQYTQAQQTR’ as an example (in which Y3 is O-GlcNAcylated).** Of note, major fragments of the tags, *i.e.*, m/z 300.130 and 503.210 for both PC- and Dde-biotin-alkyne, m/z 329.146 and 532.225 for DADPS-biotin-alkyne, and m/z 507.220 and 710.299 for Diazo-biotin-alkyne, are highlighted in *green*. O-GlcNAc site in peptide sequence is shown in *red*.

Figure 2 shows the EThcD MS/MS spectra of the tagged O-GlcNAcylated peptide having the sequence AGY(O-GlcNAc)SQGATQYTQAQQTR in which Y3 is O-GlcNAcylated, after enrichment using each of the four cleavable biotin-alkyne probes. As seen, abundant fragmentation ions (particularly c_3_^+^, c_4_^+^, z_14_^+^, and z_15_^+^) clearly annotate the O-GlcNAcylated peptide and the modified Tyr residue. Moreover, dominant fragmentation at the glycosidic linkage can be observed, as illustrated by the presence of diagnostic ion pairs of the tags (at m/z 300.130 and 503.210 for both PC- and Dde-biotin-alkyne, m/z 329.146 and 532.225 for DADPS-biotin-alkyne, and m/z 507.220 and 710.299 for Diazo-biotin-alkyne), as expected. A similar fragmentation pattern was observed in the EThcD MS/MS spectra for the other two tagged O-GlcNAcylated peptides (supplemental Fig. S1). These data suggest that all the biotin-alkyne probes worked well not only for the Ser/Thr O-GlcNAcylated peptides but also for the Tyr O-GlcNAcylated peptide. This result is in agreement with our previous finding that the chemoenzymatic labeling/click chemistry approach can enrich Tyr O-GlcNAcylated peptides in an unbiased manner (for both synthetic peptides and peptides from complex samples) (7).

We next compared the intensities of tagged O-GlcNAc peptides after enrichment using each biotin-alkyne probe (supplemental Fig. S2). Interestingly, in comparison to Dde-biotin-alkyne, the other three probes (*i.e.*, PC-biotin-alkyne, DADPS-biotin-alkyne, and Diazo-biotin-alkyne) provided higher intensities of the tagged O-GlcNAc peptides. The relatively poor performance of the Dde-biotin-alkyne probe might be due to its relative instability during sample preparation (62, 81). Of note, each O-GlcNAc peptide showed different intensities. For example, compared with PC-biotin-alkyne, DADPS-biotin-alkyne yielded significantly higher signals for Ser/Thr O-GlcNAc peptides but similar abundances for the Tyr O-GlcNAc peptide. These data suggest that the biotin-alkyne probes can unbiasedly enrich all three types of O-GlcNAc peptides. In addition, the excellent reproducibility of technical triplicates demonstrates the reliability of the analytical workflows.

### Comparison of Different Probes for O-GlcNAc Proteomics of Mouse Brain Samples

**Fig. 3:**
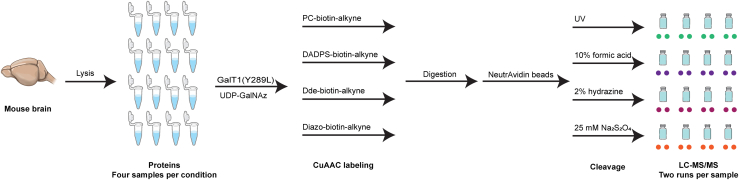
**A schematic diagram of O-GlcNAc proteomics of mouse brain samples by using four cleavable biotin-alkyne probes.** In this workflow, proteins extracted from mouse brain are subjected to GalT1(Y289L)-mediated chemoenzymatic labeling, click chemistry with cleavable biotin-alkyne probes, protein digestion with trypsin, capture with neutravidin beads, and release of tagged O-GlcNAc peptides. The released peptides are then analyzed by tandem mass spectrometry in HCD-pd-EThcD mode, with the data files analyzed by Proteome Discoverer or MaxQuant. (Four replicates were performed for each probe and the resulting peptides were injected twice for technical duplicates).

We then set out to further evaluate the performance of the cleavable bio-orthogonal probes-based methods by using complex samples. Given the relatively high O-GlcNAcylation levels and the critical physiological/pathological importance, brain samples were used to benchmark the application of the four cleavable bio-orthogonal probes for O-GlcNAc proteomics. Figure 3 illustrates the workflow, which is built on those developed for O-GlcNAc peptide samples, for brain O-GlcNAc proteomics. In brief, proteins extracted from mouse brain were subjected to GalT1(Y289L)-mediated chemoenzymatic labeling, click chemistry with cleavable biotin-alkyne probes, protein digestion with trypsin, capture with neutravidin beads, and release of tagged O-GlcNAc peptides. The released peptides were analyzed by nanoUPLC-MS/MS using 300-min gradients and HCD-pd-EThcD fragmentation, in which EThcD was triggered by the oxonium ions of HexNAc (m/z 126.055, 138.055, 144.066, 168.065, 186.076, and 204.086) and the major fragments of the tags (*i.e.*, m/z 300.130 and 503.210 for both PC- and Dde-biotin-alkyne, m/z 329.146 and 532.225 for DADPS-biotin-alkyne, and m/z 507.220 and 710.299 for Diazo-biotin-alkyne). Four replicates were performed for each probe. The resulting data files were then processed by Proteome Discoverer.

In total, more than 3 × 10^6^ MS/MS spectra were acquired, resulting in 1963 unambiguous O-GlcNAc sites from 740 O-GlcNAc proteins. A complete list of all the identified O-GlcNAc peptides, sites (including confidence scores, residues localized, and related PSMs), and proteins with unambiguous sites is shown in Supplemental Data S1. Among the cleavable biotin-alkyne probes, PC and DADPS gave comparable numbers of O-GlcNAc peptide to spectrum matches (PSMs), modified peptide sequences, unambiguous O-GlcNAc sites, and O-GlcNAc proteins with unambiguous sites (Table 1). These numbers are twice those of Dde and 4.5- to 5-fold those of Diazo. Our data suggest that PC and DADPS outperform Dde and Diazo for O-GlcNAc proteomics in general.

**Table 1:** Identifications of PSMs, unique O-GlcNAc peptides, unambiguous sites, and proteins in each dataset by PD

Identification	PC	DADPS	Dde	Diazo
MS/MS	813,230	792,185	733,768	690,778
Modified PSMs	37,609	42,048	18,746	8451
Success rate (%)	4.62	5.31	2.55	1.22
Modified PSMs activated by HCD	20,330	24,403	11,516	6186
Modified PSMs activated by EThcD	17,279T	17,645	7230	2265
HCD/EThcD ratio	1.18	1.38	1.59	2.73
Modified peptide sequences	1395	1426	878	356
Unambiguous sites	1155	1084	650	201
Proteins with unambiguous sites	566	538	308	115

All data reported is the combination of two injections from four replicates.

**Fig. 4:**
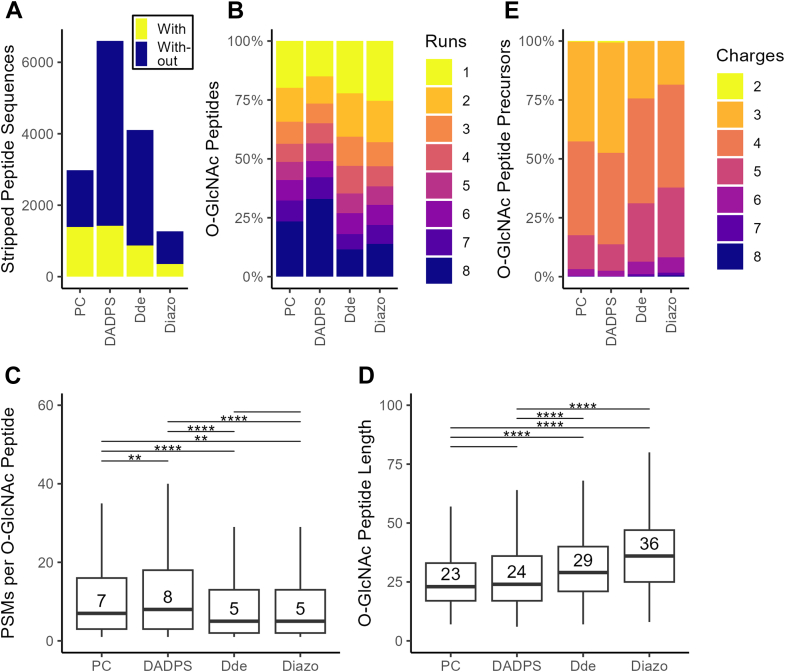
**O-GlcNAc peptides identified from mouse brain by using four cleavable biotin-alkyne probes-based O-GlcNAc proteomics.***A*, enrichment specificity of O-GlcNAc peptides. Comparison is performed in terms of stripped peptide sequence numbers with and without O-GlcNAcylation. *B*, identification frequency of O-GlcNAc peptides across eight runs for each cleavable biotin probe. *C*, distribution of total PSMs per O-GlcNAc peptide in all runs (outliers omitted). *D*, length distribution of O-GlcNAc peptides runs (outliers omitted). *E*, distribution of charge states of O-GlcNAc precursors. Statistical significance was determined using a *t* test. ∗∗*p* ≤ 0.01; ∗∗∗∗*p* ≤ 0.0001.

We next investigated the physiochemical properties of O-GlcNAc peptides identified from each biotin-alkyne probe (Fig. 4). Although a similar number of O-GlcNAc peptides (1395 *versus* 1426) was identified from PC and DADPS, the DADPS approach yielded far more non-modified peptides (Fig. 4*A*). We reasoned that the stronger hydrophobicity of the DADPS-biotin-alkyne probe might lead to non-specific binding of peptides during enrichment. An enrichment specificity of 47% was achieved for the PC approach, which is consistent with our previous work (7). Clearly, even though the four biotin-alkyne probes undergo the same processing procedures, a varied enrichment specificity (ranging from ∼22% for DADPS/Dde to ∼47% for PC) is obtained. Regarding identification reproducibility, 23% of O-GlcNAc peptides were identified by the PC approach in all eight runs, while 33% of O-GlcNAc peptides were identified by the DADPS approach (Fig. 4*B*). About 56% and 65% of the O-GlcNAc peptides were identified in at least 4 runs from the PC approach and the DADPS approach, respectively. A majority of O-GlcNAc peptides (ranging from 75% for Diazo to 85% for DADPS) were identified in at least 2 runs for all the approaches. These data further demonstrate an excellent reproducibility of the analytical workflows even for complex samples, in which DADPS and PC yield the highest reproducibility. An average of seven and eight PSMs per O-GlcNAc peptide were obtained from the PC approach and the DADPS approach, respectively (Fig. 4*C*). In contrast, five PSMs per O-GlcNAc peptide were obtained from the Dde approach and the Diazo approach. Although the PC approach and the DADPS approach contained comparative numbers of amino acids, the Dde approach and the Diazo approach yield longer peptides (up to 36 amino acids for Diazo) (Fig. 4*D*). Of note, >90% of the O-GlcNAc peptides identified from all the biotin-alkyne probes carry 3 to 5 positive charges (Fig. 4*E*). However, the Diazo approach produced a substantially higher percentage of O-GlcNAc peptides carrying more than or equal to five positive charges, which might be ascribed to the longer peptides on average. Interestingly, among O-GlcNAc peptides carrying multiple positive charges, PC yielded a slightly higher portion of peptides with five positive charges and a lower portion of peptides with three positive charges than those of DADPS (Fig. 4*E*). The slightly increased numbers of peptides carrying high positive charges could be a result of the additional positive charge of an amine group on the tag after UV cleavage. Furthermore, we investigated the overlap of O-GlcNAc peptides across four cleavable biotin probes (supplemental Fig. S3). Up to 84% (299/356) of the O-GlcNAc peptides identified from the Diazo approach were also identified by the other three probes. We reasoned that the non-overlapped peptides resulted from other probes might lead to a significant shift in the peptide length distribution. Thus, the length obtained with other probes become shorter than that of Diazo and that of the median of overlapped peptides. Lastly, we performed sequence motif analysis of all O-GlcNAc peptides identified from each probe, with largely similar patterns observed (supplemental Fig. S4). This data suggests that the four probes have comparable selectivity toward O-GlcNAc peptides/sites.

**Fig. 5:**
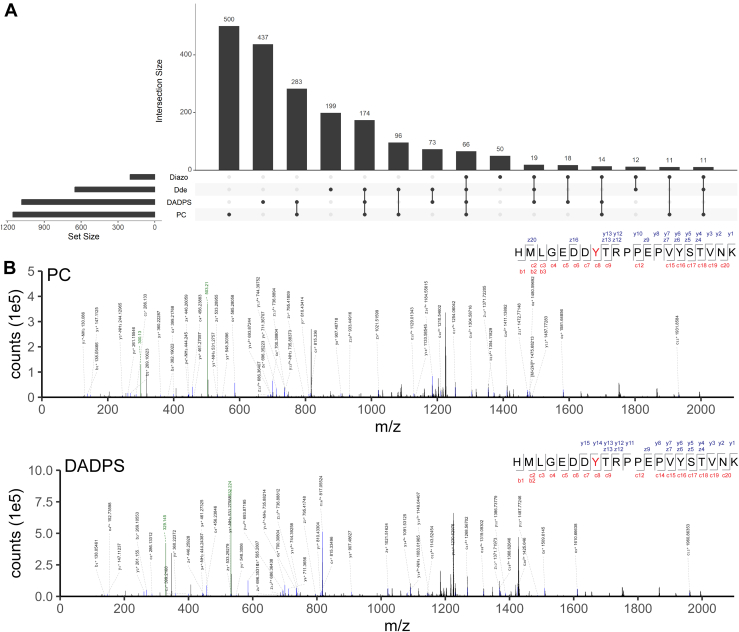
**O-GlcNAc sites identified from mouse brain, with representative mass spectra of Tyr O-GlcNAcylation illustrated.***A*, Overlap of unambiguous O-GlcNAc sites identified from mouse brain by using four cleavable biotin-alkyne probes-based O-GlcNAc proteomics. *B*, representative mass spectra of a Tyr O-GlcNAcylated peptide ^333^HMLGEDDYTRPPEPVYSTVNK^353^ of protein discs large homolog 2 (Dlg2) identified by the PC-biotin-alkyne approach (*top*) and the DADPS-biotin-alkyne approach (*bottom*). Major fragments of the tags are highlighted in *green*. O-GlcNAc site in peptide sequence is shown in *red*.

Notably, despite a comparable performance as PC and DADPS for synthetic O-GlcNAc peptides, the Diazo approach yielded fewer identifications for O-GlcNAc proteomics in brain samples, which might be due to the relatively low cleavage efficiency of diazo probes by Na_2_S_2_O_4_ as reported previously (81, 82). We then compared unambiguous O-GlcNAc sites identified using different biotin alkynes. As shown in Figure 5*A*, 1155 O-GlcNAc sites were unambiguously identified from the PC-biotin-alkyne method. Among them, 500 O-GlcNAc sites were not identified by any other biotin alkynes. The DADPS approach yielded 1084 O-GlcNAc sites, slightly less than that of PC but significantly more than that of Dde and DIAZO (which identified 650 and 201 sites, respectively). These results demonstrate that PC and DADPS are superior to Dde and Diazo for the identification of unambiguous O-GlcNAc sites. Consistent with the modified peptides identified, approximately one-fourth of the O-GlcNAc sites overlap between PC and DADPS. The representative mass spectra of a Tyr O-GlcNAcylated peptide identified using both PC- and DADPS-biotin-alkyne are shown in Figure 5*B*. The peptide ‘^333^HMLGEDDYTRPPEPVYSTVNK^353^’ from the protein discs large homolog 2 (Dlg2, also known as PSD-93/Chapsyn-110) is O-GlcNAcylated at Y340, with mass additions of 502.202 Da and 531.217 Da observed after tagging with PC and DADPS, respectively. The major fragments produced from these tags, together with the oxonium ions of the O-GlcNAc moiety (not highlighted due to space limitation), show that the peptide is O-GlcNAcylated. The fragment ions (particularly c_8_^+^, c_9_^+^, z_13_^+^, y_13_^+^, y_14_^+^) show that O-GlcNAc modifies Y340 rather than its neighboring residue T341 (Fig. 5*B*). Of note, O-GlcNAcylated Tyr sites have been detected using all biotin alkynes, with a ratio ranging from 3% to 7% among all the O-GlcNAcylated sites (supplemental Fig. S5).

The overlap of proteins corresponding to the O-GlcNAcylated sites identified across the four biotin alkynes is presented in Supplemental Fig. S6. Most proteins (61% of the 740 identified) were detected with more than one biotin alkyne, indicating that the identified O-GlcNAc modifications are concentrated in a subset of proteins of the entire mouse brain proteome.

### Construction of a Comprehensive Dataset of Brain O-GlcNAc Proteomics

**Fig. 6:**
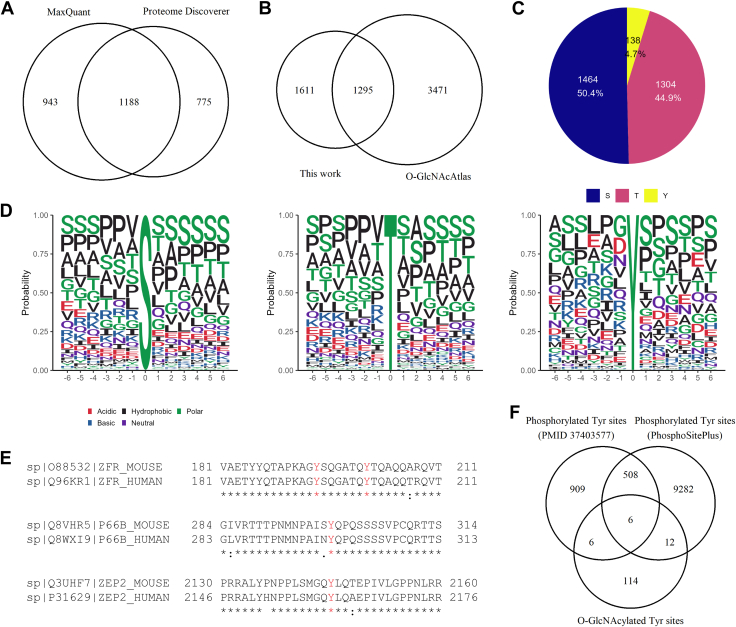
**A comprehensive dataset of unambiguous O-GlcNAc sites from mouse brain.***A*, O-GlcNAc sites identified from PD and MQ. *B*, comparison with unambiguous O-GlcNAc sites reported in mouse previously. *C*, distribution of O-GlcNAcylated residues. *D*, O-GlcNAc motif analysis. *E*, conservation of O-GlcNAcylated Tyr sites between human and mouse. Protein sequences were aligned using the Clustal Omega tool (UniProt). Symbols indicate conservation levels: “∗” fully conserved residues, “:” conservation among residues with strongly similar properties (Gonnet PAM 250 score >0.5), “.” conservation among residues with weakly similar properties (Gonnet PAM 250 score ≤0.5), and “ ” non-conserved residues. O-GlcNAc site in peptide sequence is shown in *red*. *F*, overlap of O-GlcNAcylated Tyr sites and phosphorylated Tyr sites identified from mouse brain and from other samples.

Considering that each search engine has its own distinct algorithm for peptide matching and modification localization (83), we set out to use different search engines to further improve O-GlcNAc proteome coverage. Besides PD, MaxQuant (MQ) was used to process the raw files of O-GlcNAc proteomics from all biotin alkyne probes, as we described previously (7). A list of the detailed information of unambiguous O-GlcNAc sites, including the fragmentation modes of best localized, best score, and best PEP scans, is provided in Supplemental Data S2. As shown in Figure 6*A*, besides 1188 O-GlcNAc sites unambiguously identified by both approaches, MaxQuant identified 775 additional O-GlcNAc sites. Supplemental Fig. S7 shows the representative mass spectrum of a Tyr O-GlcNAcylated peptide identified from MQ. As seen from Supplemental Fig. S7, major fragments of the tag (*i.e.*, m/z 329.145 and 532.224) and key fragment ions (including c_7_^+^, c_8_^+^, and c_9_^+^) demonstrate the O-GlcNAcylation on Y340 of the peptide ‘^333^HMLGEDDYTRPPEPVYSTVNK^353^’ from the protein discs large homolog 2 (Dlg2) after profiling with the DADPS-biotin-alkyne-based enrichment method followed by MQ analysis.

The combination of PD and MQ yielded a list of 2906 O-GlcNAc sites unambiguously assigned with high confidence (localization probability ≥75%), with details shown in Supplemental Data S3. Of note, more than 99% of the O-GlcNAc sites identified using PD had a localization probability ≥90%, and the majority of O-GlcNAc sites from MQ also exceeded this threshold (supplemental Fig. S8), suggesting high stringency of the overall localization confidence. Among all the O-GlcNAc sites unambiguously mapped, 1611 O-GlcNAc sites (55%) were newly identified, according to O-GlcNAcAtlas 4.0, which contains 4766 unambiguous O-GlcNAc sites on proteins from all types of mouse samples studied previously (Fig. 6*B*) (9). In addition to O-GlcNAcylation on many Ser/Thr sites, 138 Tyr sites were found to be O-GlcNAcylated (corresponding to 96 proteins). Among all the Tyr O-GlcNAcylation sites, 42 were identified by both PD and MQ. In addition, 40 sites and 56 sites were uniquely assigned by PD and MQ, respectively. The ratio of all O-GlcNAcylated Ser, Thr, and Tyr residues is approximately 50.4%:44.9%:4.7% (Fig. 6*C*), which is similar to our previous findings from human cells (1).

Figure 6*D* shows the flanking amino acids around the target residues. A pattern P-P-V-*S/T*-S-S/A appears to be dominant, similar to those of modified Ser/Thr observed previously (10). In contrast to O-GlcNAcylated Ser/Thr, a slightly different pattern is observed for Tyr O-GlcNAcylation. P-X-*Y*-S-P is most common for O-GlcNAcylated Tyr sites, similar to that described in a previous report using cultured human cells (7).

We evaluated the potential conservation of O-GlcNAcylated Tyr sites between mice and humans (7) (Fig. 6*E*). Out of the 96 Tyr O-GlcNAcylated proteins, 17 were also identified to be Tyr O-GlcNAcylated in cultured human cells (7), including zinc finger RNA-binding protein and transcriptional repressor p66-beta. Despite the relatively small numbers, conservation of Tyr O-GlcNAcylation is observed on a number of sites. For example, O-GlcNAcylation was found on Y194 and Y201 of the Zinc finger RNA-binding protein (Zfr) in both mice and human samples. In addition, O-GlcNAcylation on Y299 on transcriptional repressor p66-beta (Gatad2b) and Y2145 on transcription factor HIVEP2 (Hivep2) in mice was observed in the corresponding ortholog proteins in humans. The relative conservation between species indicates that O-GlcNAcylated Tyr sites is of functional importance.

To explore the potential cross-talk between Tyr O-GlcNAcylation and Tyr phosphorylation, we compared the O-GlcNAcylated Tyr sites to a recently published mouse brain phosphoproteomics study, which identified 1429 phosphorylated Tyr sites (84). 12 sites overlapped, representing 9% of all O-GlcNAcylated Tyr sites (Fig. 6*F*). This ratio was increased to 13%, when compared to a comprehensive phosphorylation database which catalogued 9808 phosphorylated Tyr sites from all mouse samples studied previously (PhosphoSitePlus (85), accessed on February 27, 2025) (Fig. 6*F*). Taken together, 24 O-GlcNAcylated Tyr sites (17%) colocalize with reported phosphorylated Tyr sites. These ratios are substantially lower than those observed in human cells (∼30.6%) upon acute treatment with Thiamet G (an OGA inhibitor) (7). This difference might be partially ascribed to the different biological conditions used, given that the O-GlcNAcylated Tyr sites in the current study were identified at physiological conditions without experimental stimulation.

**Fig. 7:**
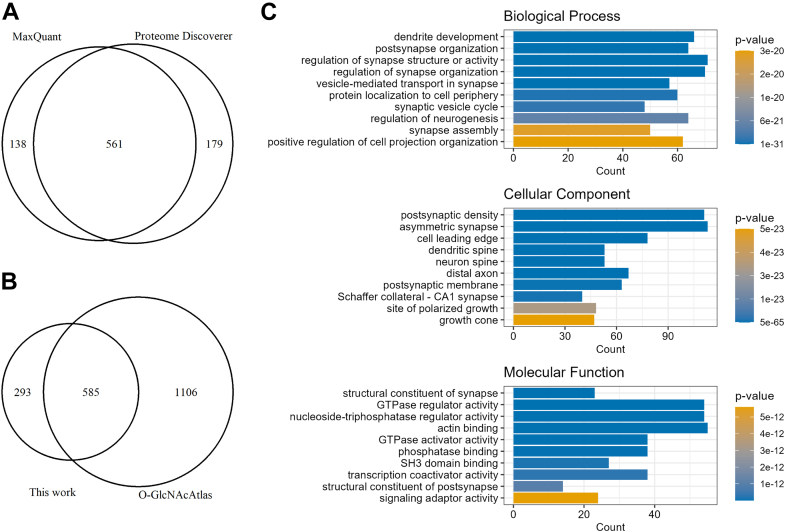
**A comprehensive dataset of O-GlcNAcylated proteins from mouse brain.***A*, proteins with unambiguous O-GlcNAcylated sites identified from PD and MQ. *B*, comparison to O-GlcNAcylated proteins with unambiguous sites reported in mouse previously. *C*, GO enrichment analysis of O-GlcNAcylated proteins with unambiguous sites.

In total, we identified 878 proteins with unambiguous O-GlcNAc sites (Fig. 7*A*). Among them, 293 proteins (33%) had not been previously identified with unambiguous O-GlcNAc sites, according to O-GlcNAcAtlas 4.0 (Fig. 7*B*). By performing subcellular localization analysis, we found that >98% of the O-GlcNAcylated proteins are localized in intracellular domains (except endoplasmic reticulum and Golgi apparatus). A GO enrichment analysis demonstrates that the 878 O-GlcNAcylated proteins are enriched in biological processes such as the dendrite development, postsynapse organization, and the regulation of synapse structure, activity and organization (Fig. 7*C*). In addition, they are highly enriched in postsynaptic density, asymmetric synapse, and associated molecular functions (Fig. 7*C*). Interestingly, a further analysis of the 96 proteins with O-GlcNAcylated Tyr sites reveals that they are also enriched in similar GO terms as those with modified Ser/Thr (supplemental Fig. S9).Of the 558 mouse kinases in the protein kinase database (86), 27 kinases were found with unambiguous O-GlcNAc sites in the current study, including glycogen synthase kinase 3 beta (Gsk3b) and Calcium/calmodulin-dependent protein kinase type IV (Camk4). Among the 96 Tyr O-GlcNAcylated proteins, 30 were also found to be Tyr phosphorylated proteins in the mouse brain phosphoproteomics dataset (84). Clearly, exploration of the roles of Ser/Thr/Tyr O-GlcNAcylation in modulating phosphorylation and the functions of kinases themselves will be an important endeavor. The detailed cross-talk mechanisms between O-GlcNAcylation and phosphorylation (particularly Tyr O-GlcNAcylation and Tyr phosphorylation) at the enzyme- and substrate modification site-specific- levels await further elucidation.

Last but not least, GlcNAcylation was identified on 342 Asn residues of 240 proteins (Supplemental Data S4). Amongst, 45% of the identified N-GlcNAc sites are annotated as known N-glycosylation sites, according to the UniProt annotation (https://www.uniprot.org/). For example, GlcNAcylation was observed on N197 of the palmitoyl-protein thioesterase 1 (PPT1) and N109 of leukocyte surface antigen CD47 (CD47) (supplemental Fig. S10). Supplemental Fig. S10*B* highlights an example of an N-GlcNAc peptide ‘^104^DAMVGNYTCEVTELSR^119^’ from CD47, a cell surface protein involved in cell-to-cell interactions. The presence of c_6_^+^ and z_11_^+^ confidently identifies the modification on N109, and the HCD fragmentation pattern (insert) shows the modification is GlcNAc rather than GalNAc. Further motif analysis of the N-GlcNAc peptides (supplemental Fig. S11) suggests strong enrichment for peptides containing the N-X-S/T sequence, similar as the classic N-glycosylation. The discovery of N-linked GlcNAc-modified peptides is somewhat unexpected, given that a single N-linked GlcNAc was identified on a number of proteins by using lectin weak-affinity chromatography-based enrichment previously (44, 45, 50). Our data suggests that the chemoenzymatic labeling-based workflow can enrich N-GlcNAc peptides, in addition to Ser/Thr/Tyr O-GlcNAc peptides. Although previous work indicated that N-GlcNAcylation can occur by cytosolic deglycosylating enzyme (endo-β-Nacetylglucosaminidase; ENGase) under normal conditions (50, 87), its functional importance in physiology and pathology remains largely unknown.

## Discussion

Protein O-GlcNAcylation plays critical roles on many proteins across species. Despite its functional importance, large scale site-specific characterization of protein O-GlcNAcylation has been a bottleneck for many years. Efficient enrichment O-GlcNAc proteins/peptides from complex samples holds promise for deep O-GlcNAc proteomics. By leveraging previous efforts, we set out to evaluate and improve existing analytical methods, aiming to establish more sensitive and robust workflows. We had previously systematically evaluated the performance of several affinity enrichment methods/materials for O-GlcNAc proteomics (74). In another study, we utilized chemoenzymatic labeling coupled with click chemistry to analyze the same set of human cell samples using the same set of instruments (7). Very encouragingly, the resulting workflow yielded unprecedented O-GlcNAc proteome depth, enabling our discovery of Tyr O-GlcNAcylation. Prompted by that work, in the present study we proposed to assess and further optimize chemoenzymatic labeling/click chemistry-based methods. Specifically, we conducted a head-to-head comparison of workflows involving four cleavable biotin-alkyne reagents for site-specific O-GlcNAc proteomics. After being evaluated using standard O-GlcNAc peptides, the methods were then benchmarked for O-GlcNAc proteomics of mouse brain samples.

By using three standard O-GlcNAc peptides, we established analytical workflows integrating GalT1 (Y289L) chemoenzymatic labeling, click chemistry with four cleavable biotin-alkyne reagents (PC, DADPS, Dde, and Diazo), and EThcD mass spectrometry for O-GlcNAc analysis (Fig. 1). We found that all the biotin-alkyne probes could be used to enrich not only Ser/Thr O-GlcNAcylated peptides but also the Tyr O-GlcNAcylated peptide, providing further evidence that the chemoenzymatic labeling/click chemistry approach can enrich all types of O-GlcNAcylated peptides in an unbiased manner (7). However, differences were observed for the resulting O-GlcNAc peptides enriched with different biotin-alkyne probes (supplemental Fig. S1). Among the probes, Dde yielded the lowest intensity of all the tagged O-GlcNAc peptides, which is likely due to its degradation during sample processing (62). By analyzing the brain lysates, we revealed that all the biotin-alkyne probes could be used to profile the O-GlcNAc proteome of complex samples, despite significant differences in coverage. In comparison to the Dde probe, PC and DADPS yield many more identifications (including more O-GlcNAc PSMs, O-GlcNAc peptides, unambiguous O-GlcNAc sites, and O-GlcNAc proteins) (Table 1). Distinct from its performance for standard O-GlcNAc peptides, the Diazo approach gave the fewest O-GlcNAc identifications. These data suggest that the PC and DADPS approaches are better choices for sensitive O-GlcNAc proteomics. Moreover, although similar reproducibility of standard O-GlcNAc peptides was achieved from workflows of all probes (supplemental Fig. S2), PC and DADPS approaches demonstrated an overall higher reproducibility of O-GlcNAc peptides from brain samples (Fig. 4*B*). From the enrichment specificity perspective, a specificity up to 47% was achieved for the PC approach, which is much higher than the other three biotin-alkyne probes (Fig. 4*A*). Even though a low specificity down to 22% was obtained from the DADPS and Dde approaches, this is still substantially higher than that of the affinity enrichment approaches (74). Collectively, our data suggest that the PC-biotin-alkyne approach, followed by the DADPS-biotin-alkyne approach, provides the highest sensitivity, reproducibility, and specificity for O-GlcNAc proteomics. Besides presenting two analytical strategies, *i.e.*, for peptide-level labeling (Fig. 1) and for protein-level labeling (Fig. 3), our work provides invaluable insights into the technical aspects of O-GlcNAc proteomics and will help guide the selection of appropriate methods for O-GlcNAc proteomics by others. Of note, we employed HCD-pd-EThcD in which oxonium ions from HCD were used to trigger EThcD fragmentation in our study, with the generated HCD and EThcD spectra searched together in a combined workflow during data processing. We noticed that although the vast majority of O-GlcNAc sites were identified by spectra based on EThcD only or both HCD and EThcD, a small portion (<10%) was exclusively contributed by HCD mass spectra. Despite a high localization threshold (≥0.75) being used, careful annotation of such sites should be taken. Efforts on the development of more advanced mass spectrometric fragmentation techniques and site-scoring algorithms would facilitate improved site localization in O-GlcNAc proteomics studies.

We exemplified the application of the O-GlcNAc proteomics workflows to the profiling of O-GlcNAcylated proteins in mouse brain lysates, as brain (61, 63, 68) and the synaptosome (43, 45) contain proteins heavily modified by O-GlcNAcylation. Using mouse brain lysates as a complex sample, we generated a comprehensive dataset of protein O-GlcNAcylation. In total, 2906 O-GlcNAc sites were unambiguously mapped on 878 proteins. Remarkably, this is several-fold higher than the numbers of O-GlcNAc sites identified from mouse brain samples previously (61, 63, 68). To our knowledge, this represents not only the biggest dataset of an individual project on brain samples, but also the biggest dataset of O-GlcNAc sites reported in an individual study of any samples to date. Besides mapping many known O-GlcNAc sites, 1611 O-GlcNAc sites were newly identified in this study. For example, microtubule-associated protein 2 (MAP-2), a dendritically enriched protein and marker of synaptic plasticity (88), was previously reported to be an O-GlcNAc protein, with a number of sites identified (45, 49, 68). In addition to mapping several known sites, we unambiguously identified several new O-GlcNAc sites, including S361 (which was reported previously as an ambiguous site (49)), S496, and S1057. Of note, although our dataset is of unprecedented depth, there is still room to further expand the catalog of O-GlcNAcylated proteins/sites. For example, O-GlcNAcylation on S705, S714, and T719 on the microtubule-associated protein tau was identified by our strategy, whereas two O-GlcNAcylated sites, S692 and T705 were identified from tau-enriched samples and mouse hippocampus samples (49, 89). Thus, instead of using the whole brain lysates, other sample preparation methods such as sub-region fractionation or immunoprecipitation will certainly help increase the identification of O-GlcNAcylated sites/proteins. We anticipate that the substantially expanded dataset of O-GlcNAcylated sites/proteins in the brain will spur more interest in probing the roles of O-GlcNAcylation in the brain. Arguably, further functional studies on protein O-GlcNAcylation will provide a deeper understanding of brain physiology and facilitate elucidation of the etiology of neurogenerative diseases such as Alzheimer’s disease and Parkinson’s disease.

Finally, our dataset of mouse brain O-GlcNAc proteomics provides invaluable insights into the biology of protein O-GlcNAcylation, particularly Tyr O-GlcNAcylation. Our previous work identified O-GlcNAcylation on 121 Tyr sites on proteins in cultured human cancer cells stimulated with thiamet G (an inhibitor of OGA) (7). However, several questions remained, *e.g.*, 1) it was unclear whether Tyr O-GlcNAcylation existed on endogenous proteins without any stimulation, and 2) it was unknown whether Tyr O-GlcNAcylation would be a common phenomenon in other types of samples. Very encouragingly, our mouse brain O-GlcNAc proteomics datasets have offered some valuable hints. Firstly, 138 Tyr sites were found O-GlcNAcylated from mouse brain, suggesting that Tyr O-GlcNAcylation can be detected without external stimulation, and Tyr O-GlcNAcylation does exist in sample sources other than human cancer cells. Based upon all these findings, we argue that Tyr O-GlcNAcylation is a widespread modification on many proteins in multiple species. Secondly, comparing the identified Tyr O-GlcNAcylation sites between human and mouse samples allows us to explore the potential conservation of Tyr O-GlcNAcylation across species. Although the datasets are still relatively small and the sample types are not strictly comparable, we do observe conservation of some Tyr O-GlcNAcylation sites on proteins. The cross-species conservation of Tyr O-GlcNAcylation sites further indicates that Tyr O-GlcNAcylation has a regulatory role. Thirdly, our work enables a better understanding of other key features of Tyr O-GlcNAcylation, such as its percentage distribution and site-specific cross-talk with Tyr phosphorylation. Amongst the O-GlcNAc-modified residues, the ratio of O-GlcNAcylated Tyr appears to be relatively small (4–5%). Moreover, O-GlcNAcylated Tyr sites and phosphorylated Tyr sites do not seem to overlap overwhelmingly (<15%) in basal conditions, displaying certain agreement with that observed between O-GlcNAcylated Ser/Thr sites and phosphorylated Ser/Thr sites (45). It will be intriguing to see whether there is a higher degree of colocalization (*i.e.*, reciprocal competition) between Tyr O-GlcNAcylation and Tyr phosphorylation in stimulated conditions, as shown in our previous report (7). In addition, besides many serine/threonine kinases, several tyrosine kinases, including tyrosine-protein kinase ABL2 (Abl2) and tyrosine kinase non-receptor protein 2 (activated CDC42 kinase 1, Tnk2), are themselves O-GlcNAcylated. Further exploration of the roles of O-GlcNAcylation on tyrosine kinases' function will offer new insights into the cross-talk at the enzyme level, as well as the intracellular network mediated by Ser/Thr/Tyr O-GlcNAcylation and Ser/Thr/Tyr phosphorylation.

In conclusion, we developed chemoenzymatic labeling/click chemistry-based analytical workflows for O-GlcNAc proteomics, focusing on assessing the performance of four cleavable biotin-alkyne reagents (PC-biotin-alkyne, DADPS-biotin-alkyne, Dde-biotin-alkyne, and Diazo-biotin-alkyne). Besides standard O-GlcNAc peptides, mouse brain samples were used to validate the analytical merits of these reagents. Our data suggest that workflows using PC-biotin-alkyne and DADPS-biotin-alkyne outperform the others regarding sensitivity, reproducibility, and specificity for O-GlcNAc proteomics. The analytical workflows established and the evaluations performed will help guide the selection of analytical workflows for site-specific and systems-scale O-GlcNAc proteomics studies. By benchmarking their applications to mouse brain samples, we have generated the largest dataset of brain O-GlcNAc proteomics to date, which provides not only a reference for further studying O-GlcNAcylation in the brain but also critical insight into Tyr O-GlcNAcylation. Taken together, our data provides both invaluable insights into the technical aspects of O-GlcNAc proteomics and additional understanding of O-GlcNAcylation biology (particularly Tyr O-GlcNAcylation). We anticipate that our work will likely contribute to the acceleration of O-GlcNAc proteomics research, creating instrumental tools to interrogate functional roles of protein O-GlcNAcylation in a wide range of biomedical settings.

## Data Availability

This article contains supplemental data, including all validated data reported in tabular format. The mass spectrometry proteomics data, including raw, .msf, and annotated/indexed glycopeptide spectra, have been deposited to the ProteomeXchange Consortium via the PRIDE (90) partner repository with the dataset identifier PXD063995 and 10.6019/PXD063995. Annotated data used for the generation of figures, as well as all codes utilized for generating figures and processing data, are available upon request to the corresponding author.

## Supplemental Data

This article contains supplemental data (7, 53, 74).

## Conflict of interest

The authors declare that they have no conflicts of interest with the contents of this article.
