# Supplementary material for: Comprehensive Evaluation of Cleavable Bioorthogonal Probes for Site-Specific O-GlcNAc Proteomics
Source: Mol Cell Proteomics. 2025 Aug 28;24(10):101064. doi: 10.1016/j.mcpro.2025.101064 (PMC12506516; doi:10.1016/j.mcpro.2025.101064)
Supplement: Supplemental Figures and Table [file mmc5.docx]

*Supplemental Data for:*

**Comprehensive evaluation of cleavable bioorthogonal probes for site-specific O-GlcNAc proteomics**

**Chunyan Hou,^1^ Hemeng Zhang,^1^ Jingtao Deng,^1^ Xiaoxin Wang,**^2^ **Stephen Byers,^1^ Moshe Levi,^2^ Daniel TS Pak,^3^ Kelley W. Moremen,^4^ Huadong Pei,^1^ Gerald W Hart,^4^ Junfeng Ma^1,*^**

^1^ Department of Oncology, Lombardi Comprehensive Cancer Center, Georgetown University Medical Center, Washington DC 20007, USA

^2^ Department of Biochemistry and Molecular & Cellular Biology, Georgetown University Medical Center, Washington DC 20007, USA

^3^ Department of Pharmacology and Physiology, Georgetown University Medical Center, Washington DC 20007, USA

^4^ Complex Carbohydrate Research Center, University of Georgia, Athens, GA 30602, USA

^*^ To whom correspondence should be addressed: Tel: +1-202-6873802; e-mail: junfeng.ma@georgetown.edu

**Running title**: Evaluation of bioorthogonal probes for O-GlcNAc proteomics

***Table of Contents***

| **Description** | **Page No.** |
| --- | --- |
| **Supplemental Fig. S1.** Representative mass spectra of synthetic O-GlcNAc peptides after enrichment. | S-3 to S-4 |
| **Supplemental Fig. S2.** Intensities of standard O-GlcNAc peptides after enrichment. | S-5 |
| **Supplemental Fig. S3.** Overlap of O-GlcNAc peptides identified from mouse brain by using biotin-alkyne probes-based O-GlcNAc proteomics. | S-6 |
| **Supplemental Fig. S4.** Sequence motifs of O-GlcNAcylated peptides identified by using different probes. | S-7 |
| **Supplemental Fig. S5.** Distribution of O-GlcNAcylated S/T/Y residues identified by using different probes. | S-8 |
| **Supplemental Fig. S6.** Overlap of O-GlcNAc proteins with unambiguous sites identified from mouse brain by using biotin-alkyne probes-based O-GlcNAc proteomics. | S-9 |
| **Supplemental Fig. S7**. Representative mass spectrum of a Tyr O-GlcNAcylated peptide identified from MaxQuant. | S-10 |
| **Supplemental Fig. S8.** Localiation probability distribution of O-GlcNAc sites identified using Proteome Discoverer and MaxQuant | S-11 |
| **Supplemental Fig. S9**. GO enrichment analysis of Tyr O-GlcNAcylated proteins. | S-12 |
| **Supplemental Fig. S10**. Representative EThcD mass spectra of N-GlcNAc peptides from palmitoyl-protein thioesterase 1 and leukocyte surface antigen CD47. | S-13 |
| **Supplemental Fig. S11**. Motif analysis of identiffied N-GlcNAc peptides. | S-14 |
| **Supplemental Table S1.** Comparison of O-GlcNAc proteomics methods for PANC-1 cell lysates. | S-15 |
| **Supplemental Data S1.** Lists of O-GlcNAc peptides, unambiguous sites, and proteins identfied from different probes by Proteome Discoverer. |  |
| **Supplemental Data S2.** Lists of unambiguous O-GlcNAc sites identfied from different probes by MaxQuant. |  |
| **Supplemental Data S3.** Summary of all unambiguous O-GlcNAc sites identified. |  |
| **Supplemental Data S4**. List of unambiguous N-GlcNAc sites identified. |  |







**Supplemental Fig. S1.** Representative mass spectra of synthetic O-GlcNAc peptides after enrichment (with O-GlcNAc sites shown in red).


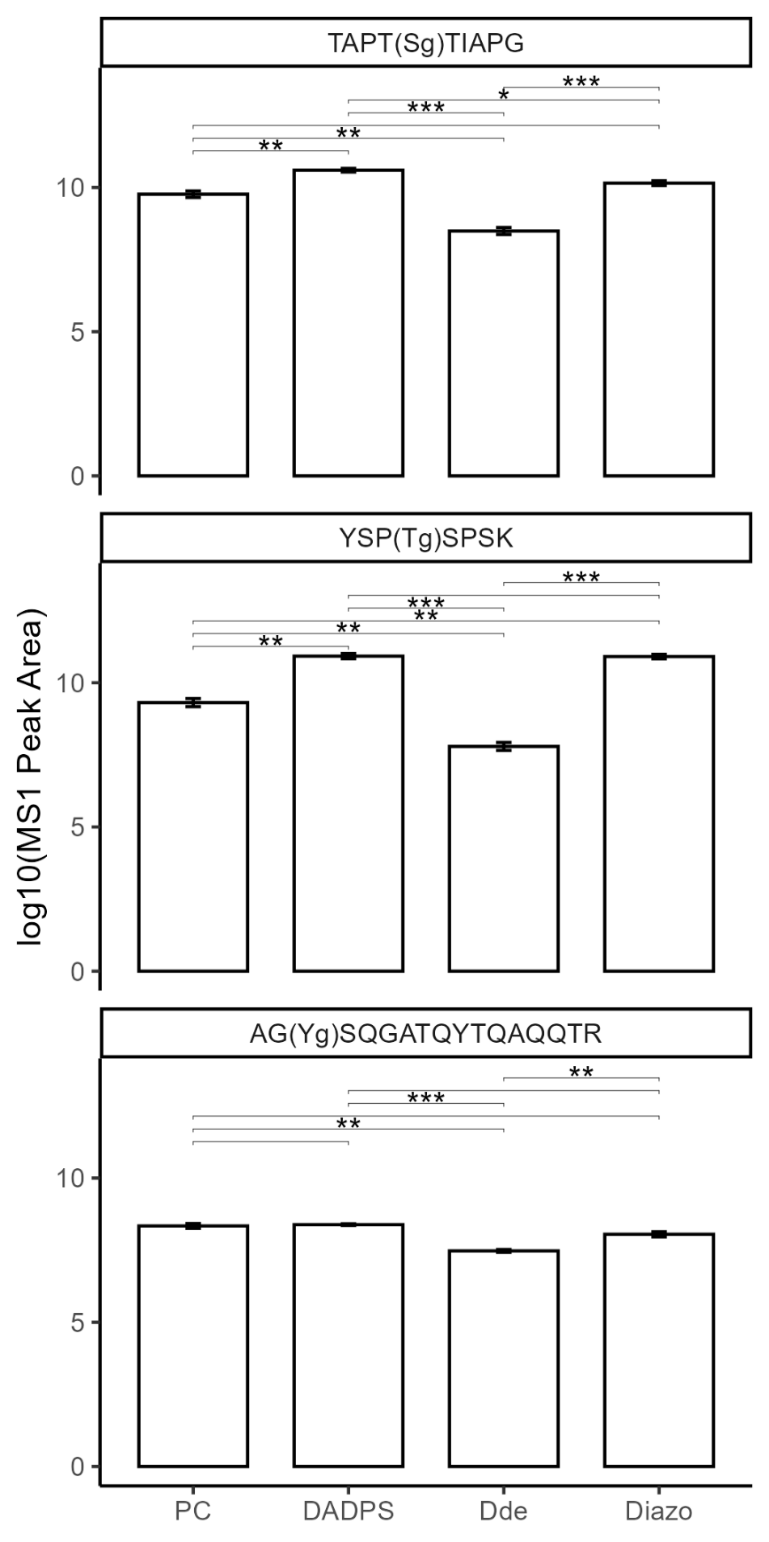


**Supplemental Fig. S2.** Intensities of standard O-GlcNAc peptides after enrichment. Sg/Tg/Yg indicates O-GlcNAcylated Ser, Thr, or Tyr in the peptide sequence. Error bars represent standard error. Statistical significance was determined using a t-test. * p ≤ 0.05; ** p ≤ 0.01; *** p ≤ 0.001.


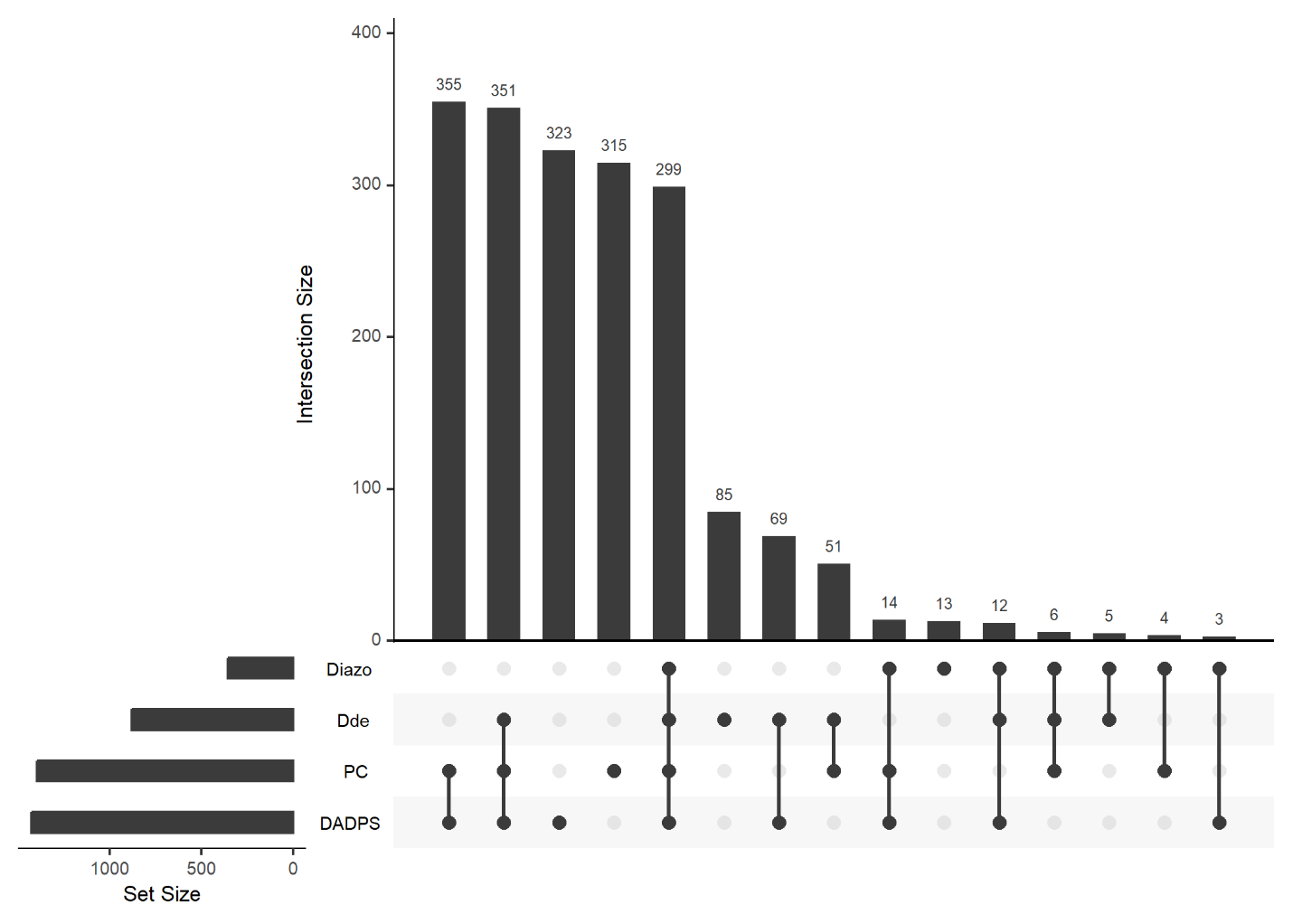


**Supplemental Fig. S3.** Overlap of O-GlcNAc peptides identified from mouse brain by using biotin-alkyne probes-based O-GlcNAc proteomics.


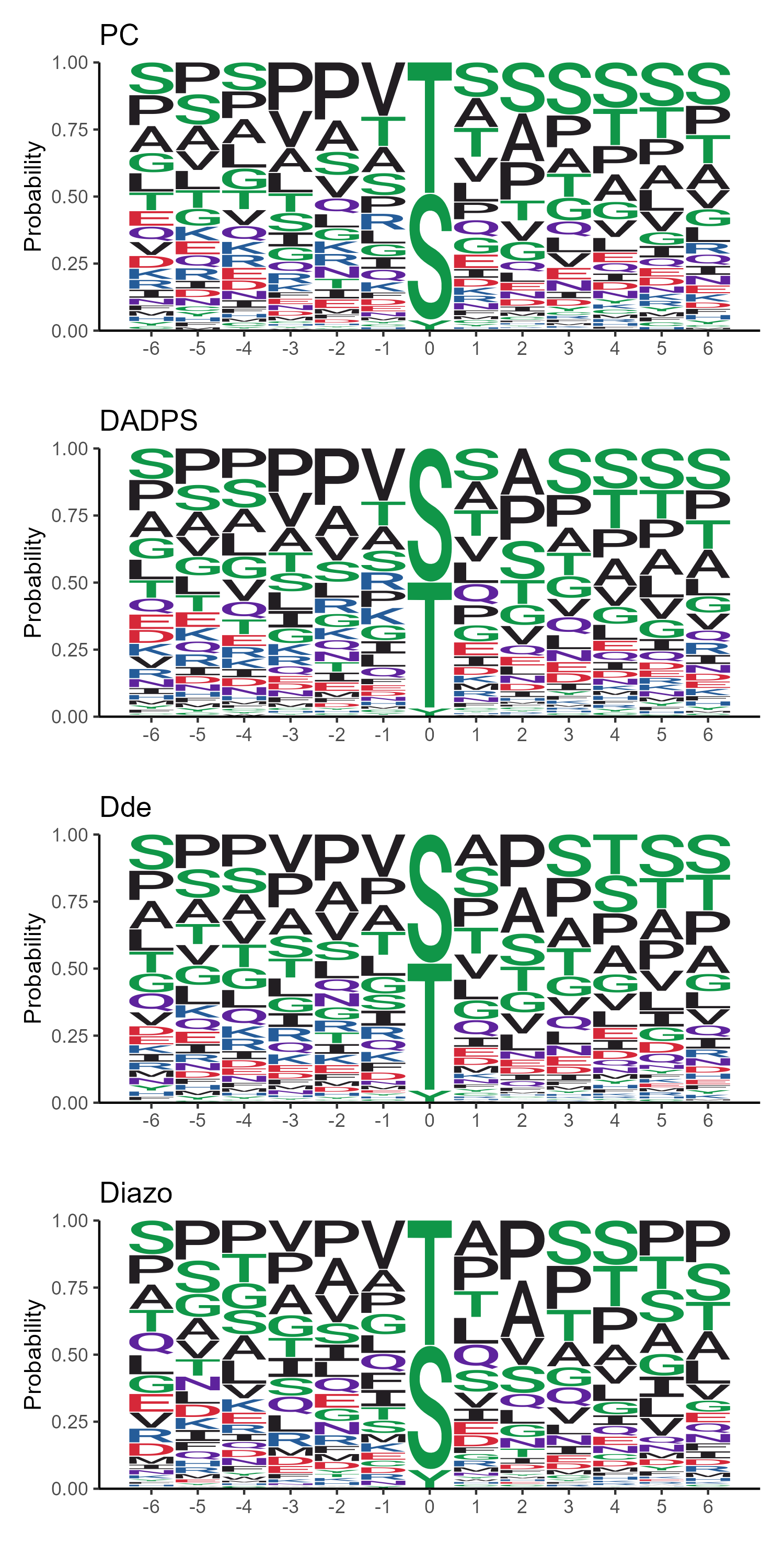


**Supplemental Fig. S4.** Sequence motifs of O-GlcNAcylated peptides identified by using different probes.


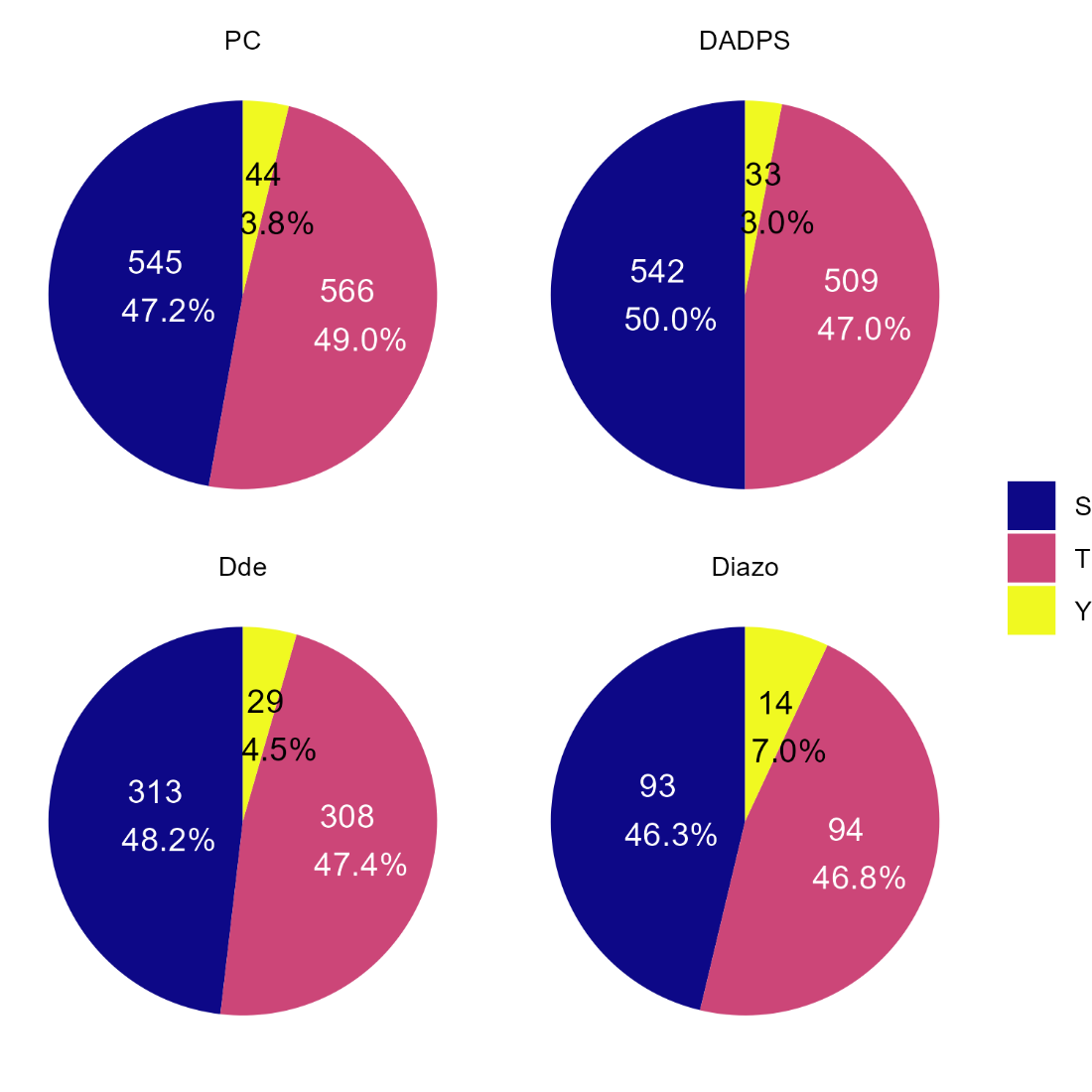


**Supplemental Fig. S5.** Distribution of O-GlcNAcylated S/T/Y residues identified by using different probes.


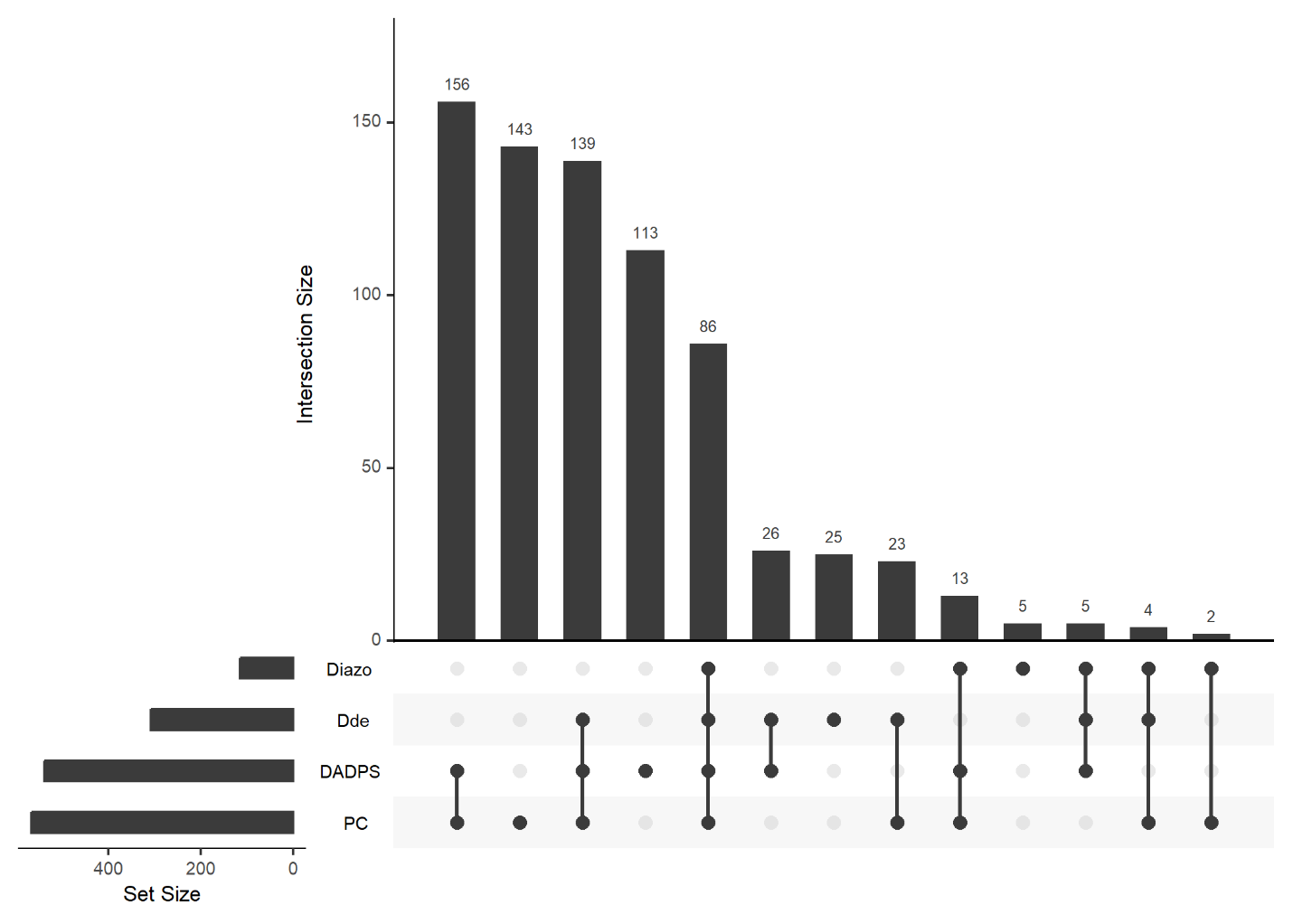


**Supplemental Fig. S6.** Overlap of O-GlcNAc proteins with unambiguous sites identified from mouse brain by using biotin-alkyne probes-based O-GlcNAc proteomics.


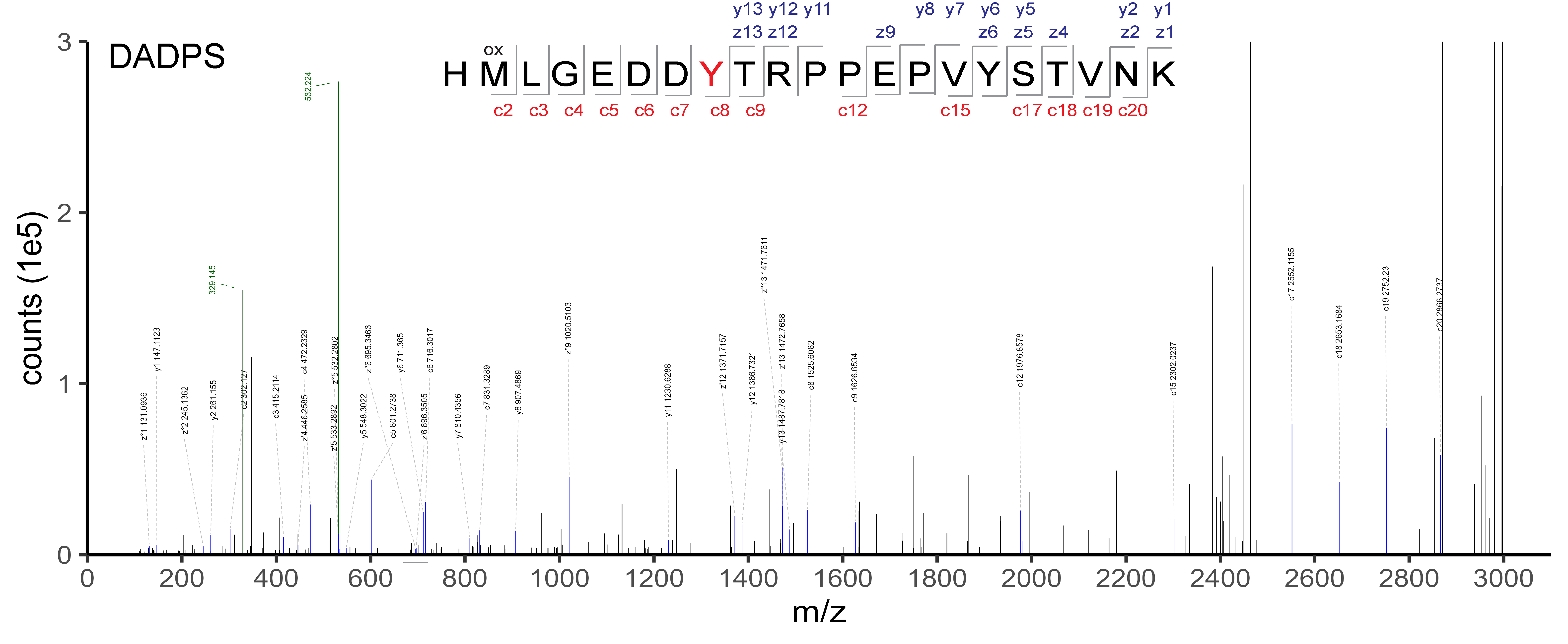


**Supplemental Fig. S7.** Representative mass spectrum of a Tyr O-GlcNAcylated peptide identified from MaxQuant analysis. A Tyr O-GlcNAcylated peptide ‘^333^HMLGEDDYTRPPEPVYSTVNK^353^’ of disks large homolog 2 (Dlg2) was identified by the DADPS-biotin-alkyne approach after MaxQuant analysis. Of note, major fragments of the tags (i.e., m/z 329.145 and 532.224) are highlighted in green and the O-GlcNAc site in peptide sequence is shown in red.

**
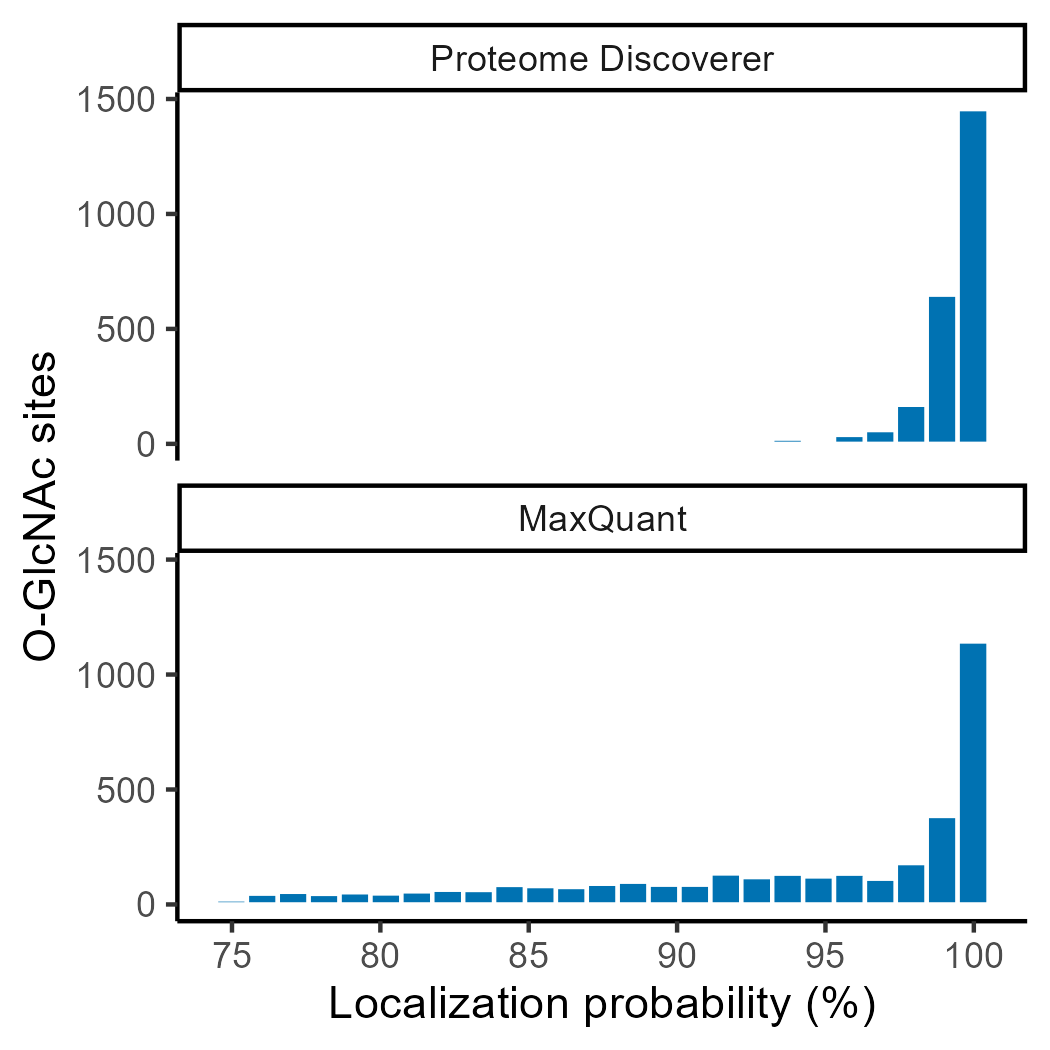
**

**Supplemental Fig. S8.** Localiation probablity distribution of O-GlcNAc sites identified using Proteome Discoverer and MaxQuant.


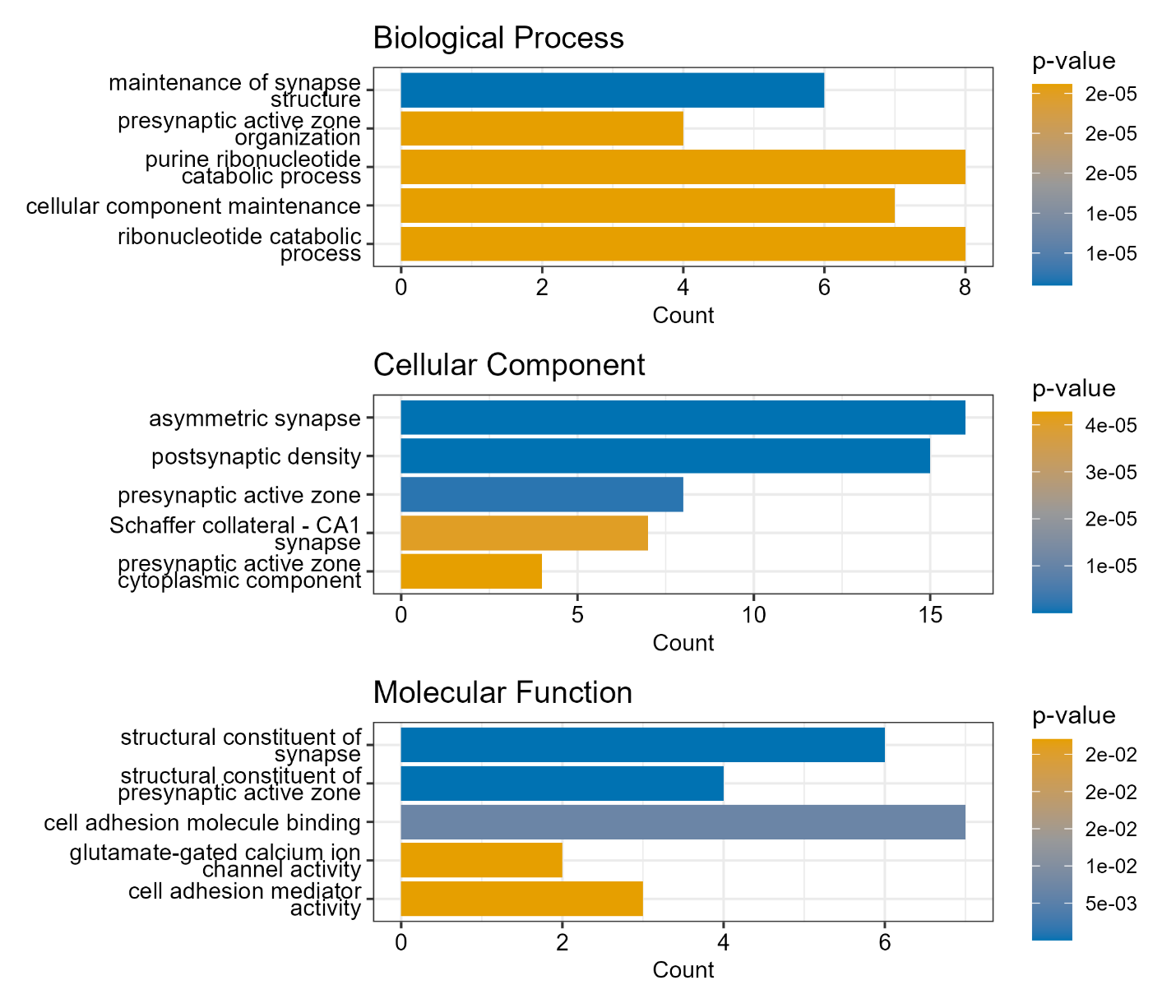


**Supplemental Fig. S9**. GO enrichment analysis of Tyr O-GlcNAcylated proteins.

**
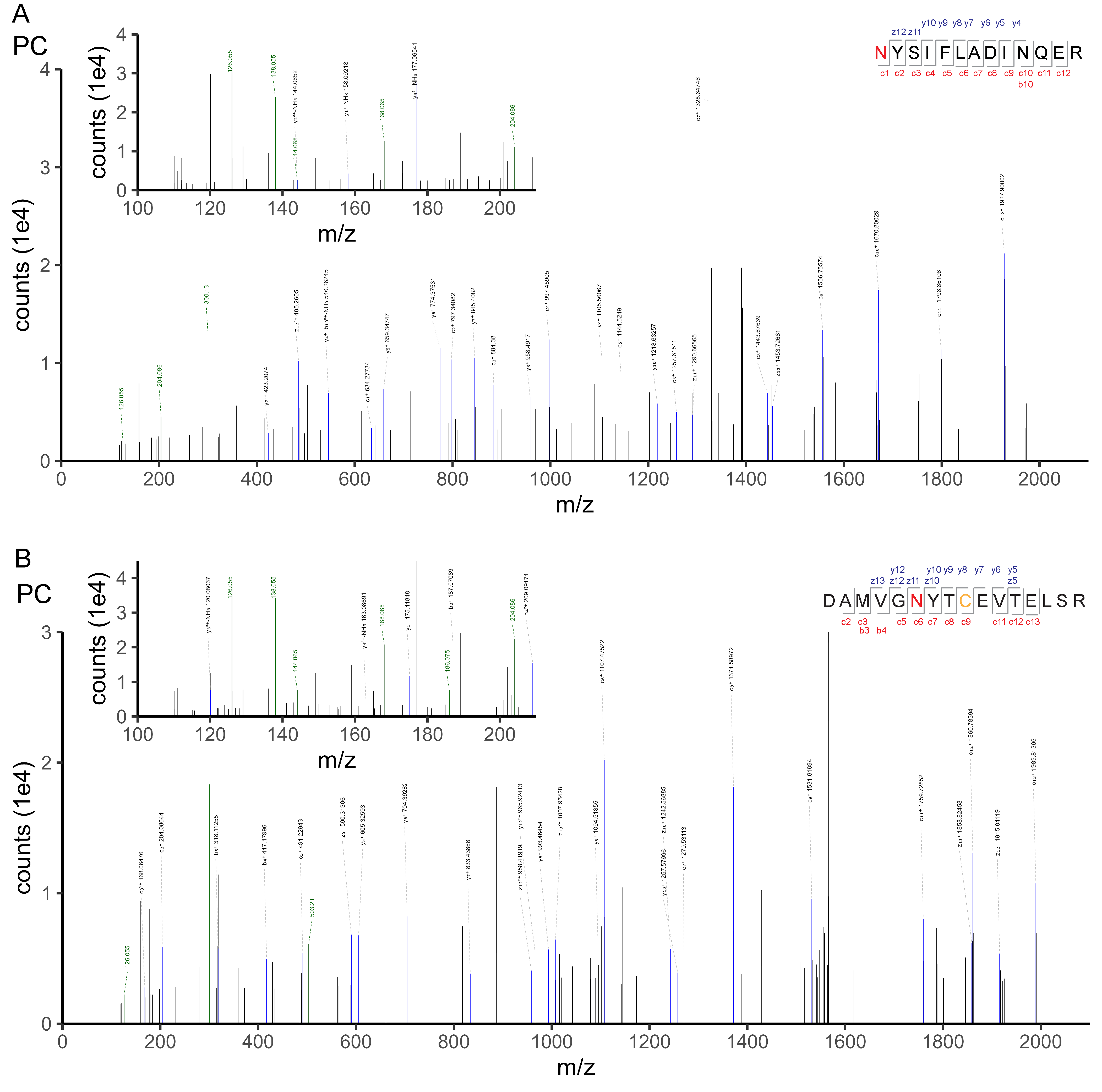
**

**Supplemental Fig. S10**. Representative EThcD mass spectra of N-GlcNAc peptides from palmitoyl-protein thioesterase 1 (A) and leukocyte surface antigen CD47 (B). GlcNAc modified Asn reside in the peptide sequence is shown in red, with carbamidomethylated Cys shown in yellow. Of note, major fragments of the tag (i.e., m/z 503.21 and 300.13) and the oxonium ions of HexNAc are labeled in green in the mass spectra. The insert in each figure illustrates the corresponding HCD spectrum of the glycopetide, showing that the modification is GlcNAc.


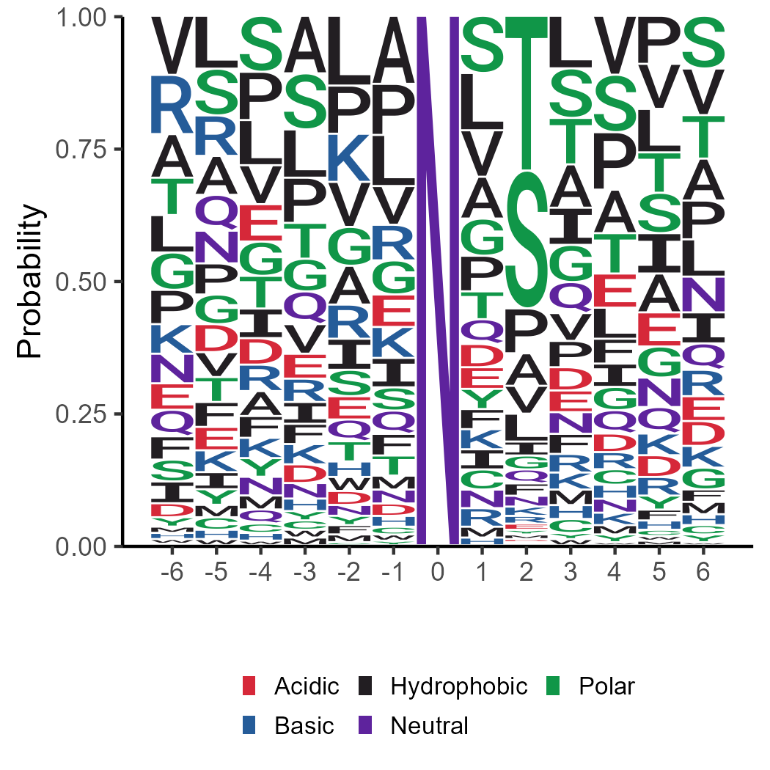


**Supplemental Fig. S11**. Motif analysis of identified N-GlcNAc peptides.

**Supplemental Table S1**. Comparison of O-GlcNAc proteomics methods for PANC-1 cell lysates.

| Input | Enrichment methods/materials | High pH RPLC fractionation | Mass spectrometry | Data analysis software | Unambiguous O-GlcNAc sites identified | Ref. |
| --- | --- | --- | --- | --- | --- | --- |
| 10 mg | PTMScan O-GlcNAc [GlcNAc-S/T] motif kit (Ab-beads) | 10 fractions | HCD-pd-EThcD on Orbitrap Lumos | Proteome Discoverer 2.4 | 62 | ^1^ |
| 10 mg | Lectin AANL6-beads | 10 fractions | HCD-pd-EThcD on Orbitrap Lumos | Proteome Discoverer 2.4 | 69 | ^1^ |
| 10 mg | OGA mutant (CpOGA^D298N^) beads | 10 fractions | HCD-pd-EThcD on Orbitrap Lumos | Proteome Discoverer 2.4 | 68 | ^1^ |
| 5 mg | nitro-oxide-grafted nanospheres | 5 fractions | HCD-pd-EThcD on Orbitrap Lumos | Proteome Discoverer 2.4 | 230 | ^2^ |
| 2 mg | GalT1 (Y289L)-based chemoenzymatic labeling and PC-biotin-alkyne click chemistry-based enrichment | None | HCD-pd-EThcD on Orbitrap Lumos | Proteome Discoverer 2.4 | 1336 | ^3^ |

**References:**

(1) Hou, C.; Wu, C.; Wu, Z.; Cheng, Y.; Li, W.; Sun, H.; Ma, J. Systematic Evaluation of Affinity Enrichment Methods for O-GlcNAc Proteomics. *J. Proteome Res.* **2024**, *23* (10), 4422–4432. https://doi.org/10.1021/acs.jproteome.4c00388.

(2) Wu, C.; Shi, S.; Hou, C.; Luo, Y.; Byers, S.; Ma, J. Design and Preparation of Novel Nitro-Oxide-Grafted Nanospheres with Enhanced Hydrogen Bonding Interaction for O-GlcNAc Analysis. *ACS Appl. Mater. Interfaces* **2022**, *14* (42), 47482–47490. https://doi.org/10.1021/acsami.2c15039.

(3) Hou, C.; Deng, J.; Wu, C.; Zhang, J.; Byers, S.; Moremen, K. W.; Pei, H.; Ma, J. Ultradeep O-GlcNAc Proteomics Reveals Widespread O-GlcNAcylation on Tyrosine Residues of Proteins. *Proc. Natl. Acad. Sci.* **2024**, *121* (47), e2409501121. https://doi.org/10.1073/pnas.2409501121.
